# Effectiveness of Structured Care Coordination for Children With Medical Complexity: The Complex Care for Kids Ontario (CCKO) Randomized Clinical Trial

**DOI:** 10.1001/jamapediatrics.2023.0115

**Published:** 2023-03-20

**Authors:** Eyal Cohen, Samantha Quartarone, Julia Orkin, Myla E. Moretti, Abby Emdin, Astrid Guttmann, Andrew R. Willan, Nathalie Major, Audrey Lim, Sanober Diaz, Lisa Osqui, Joanna Soscia, Longdi Fu, Sima Gandhi, Anna Heath, Nora Fayed

**Affiliations:** 1 Child Health Evaluative Sciences, The Hospital for Sick Children, Toronto, Ontario, Canada; 2 Institute of Health Policy, Management and Evaluation, University of Toronto, Toronto, Ontario, Canada; 3 Division of Paediatric Medicine, The Hospital for Sick Children, Toronto, Ontario, Canada; 4 Department of Paediatrics, University of Toronto, Toronto, Ontario, Canada; 5 ICES, Toronto, Ontario, Canada; 6 Edwin S.H. Leong Centre for Healthy Children, University of Toronto, Toronto, Ontario, Canada; 7 Clinical Trials Unit, Ontario Child Health Support Unit, The Hospital for Sick Children, Toronto, Ontario, Canada; 8 Dalla Lana School of Public Health, University of Toronto, Toronto, Ontario, Canada; 9 Sunnybrook Hospital, Toronto, Ontario, Canada; 10 Department of Paediatrics, Children’s Hospital of Eastern Ontario, University of Ottawa, Ottawa, Ontario, Canada; 11 Department of Pediatrics, Hamilton Health Sciences Centre, McMaster University, Hamilton, Ontario, Canada; 12 Provincial Council for Maternal and Child Health, Toronto, Ontario, Canada; 13 Lawrence M. Bloomberg Faculty of Nursing, University of Toronto, Toronto, Ontario, Canada; 14 Division of Biostatistics, Dalla Lana School of Public Health, University of Toronto, Toronto, Ontario, Canada; 15 Department of Statistical Science, University College London, London, United Kingdom; 16 School of Rehabilitation Therapy, Queen’s University, Kingston, Ontario, Canada

## Abstract

**Importance:**

Children with medical complexity (CMC) have chronic conditions and high health needs and may experience fragmented care.

**Objective:**

To compare the effectiveness of a structured complex care program, Complex Care for Kids Ontario (CCKO), with usual care.

**Design, Setting, and Participants:**

This randomized clinical trial used a waitlist variation for randomizing patients from 12 complex care clinics in Ontario, Canada, over 2 years. The study was conducted from December 2016 to June 2021. Participants were identified based on complex care clinic referral and randomly allocated into an intervention group, seen at the next available clinic appointment, or a control group that was placed on a waitlist to receive the intervention after 12 months.

**Intervention:**

Assignment of a nurse practitioner–pediatrician dyad partnering with families in a structured complex care clinic to provide intensive care coordination and comprehensive plans of care.

**Main Outcomes and Measures:**

Co-primary outcomes, assessed at baseline and at 6, 12, and 24 months postrandomization, were service delivery indicators from the Family Experiences With Coordination of Care that scored (1) coordination of care among health care professionals, (2) coordination of care between health care professionals and families, and (3) utility of care planning tools. Secondary outcomes included child and parent health outcomes and child health care system utilization and cost.

**Results:**

Of 144 participants randomized, 141 had complete health administrative data, and 139 had complete baseline surveys. The median (IQR) age of the participants was 29 months (9-102); 83 (60%) were male. At 12 months, scores for utility of care planning tools improved in the intervention group compared with the waitlist group (adjusted odds ratio, 9.3; 95% CI, 3.9-21.9; *P* < .001), with no difference between groups for the other 2 co-primary outcomes. There were no group differences for secondary outcomes of child outcomes, parent outcomes, and health care system utilization and cost. At 24 months, when both groups were receiving the intervention, no primary outcome differences were observed. Total health care costs in the second year were lower for the intervention group (median, CAD$17 891; IQR, 6098-61 346; vs CAD$37 524; IQR, 9338-119 547 [US $13 415; IQR, 4572-45 998; vs US $28 136; IQR, 7002-89 637]; *P* = .01).

**Conclusions and Relevance:**

The CCKO program improved the perceived utility of care planning tools but not other outcomes at 1 year. Extended evaluation periods may be helpful in assessing pediatric complex care interventions.

**Trial Registration:**

ClinicalTrials.gov Identifier: NCT02928757

## Key Points

**Question:**

What is the effectiveness of a pediatric complex care program that assigned a nurse practitioner–pediatrician dyad partnering with families to provide intensive care coordination and comprehensive plans of care in Ontario, Canada, compared with usual care?

**Findings:**

This randomized clinical trial found that patients randomized to receive complex care services had increased scores for utility of care planning tools at 1 year but no significant differences in other care coordination, child, parent, and health care system utilization outcome measures compared with those who were waitlisted for 1 year. Some outcome differences between the groups, including decreased overall cost, were observed at 2 years.

**Meaning:**

This complex care program improved the perceived utility of care planning tools but not other outcomes at 1 year.

## Teaser

This randomized clinical trial uses a waitlist design to assess the effects of a pediatric complex care program on the experiences of children with medical complexity and their parents or caregivers.

## Introduction

Children with medical complexity (CMC) have been defined as those with complex chronic conditions requiring specialized care, with substantial health care needs, functional limitations, and high health resource utilization.^1^ CMC and their families interact with multiple services along the care continuum and often experience substantial gaps in care due to poor care coordination,^2,3,4^ disjointed services,^5^ multiple prolonged and potentially preventable hospitalizations,^6^ receiving care from multiple clinicians,^7^ higher risk of medication order errors,^8,9^ and extraordinary stress on parents and caregivers.^10,11^ The consequences include social isolation,^12,13^ poor caregiver health,^11,14,15^ fragmentation between family caregivers and health care professionals,^16,17^ and profound financial and social hardships.^17,18^

In an ideal care delivery model for CMC, a clinician who is familiar with the child and family leads team-based coordinated care^19,20^ that addresses the comprehensive needs of the child and family, facilitates the creation of proactive plans based on family and child goals, and ensures the timely treatment of urgent acute health issues and multidisciplinary shared decision-making.^21^ Coordinated models of care have been launched widely, but rigorous evaluation has been limited by inadequate control groups, small sample sizes, single-center (mostly hospital-based) designs, and lack of a comprehensive outcome measurement framework.^22^ Among the relatively few published randomized clinical trials (RCTs), 3 parallel-group RCTs described a decrease in both rates of severe illness and health care costs,^23,24,25^ while another cluster RCT reported increased costs with no change in functional status or hospital-based utilization.^26^

Complex Care for Kids Ontario (CCKO) was launched in Ontario, Canada (population approximately 14.5 million), by its single-payer funder of health care services (the Ministry of Health). The goals of the CCKO intervention were to improve care continuity and coordination, facilitate communication and information sharing among the family and members of the care team, deliver care closer to home, and reduce health care system costs through more integrated health care delivery. The core components of CCKO included assignment of a nurse practitioner, partnering with a pediatrician to lead care; development and dissemination of a complex care plan; and care coordination across locations, the care continuum, and service systems.^27,28,29^ A description of CCKO has been published previously highlighting specific elements of care coordination and planning.^30,31,32^ The aim of this study was to compare the effectiveness of the CCKO intervention with usual care.

## Methods

### Trial Design, Setting, and Participants

The CCKO trial used a waitlist variation of a pragmatic RCT design, as detailed in the published protocol^31^ and in Supplement 1 and Supplement 4, and is reported according to the Consolidated Standards of Reporting Trials (CONSORT) reporting guidelines.^33^ The study received ethics approval from The Hospital for Sick Children Research Ethics Board.

The waitlist approach involved rolling out the intervention over time. Participants were randomized into 2 groups to receive the intervention at different time points. The intervention group received coordinated care at the next available appointment, and the waitlist group received the intervention after 12 months. This study design was chosen to address the operational need for staggered rollout of the intervention to all eligible children, while minimizing risk of bias by retaining the design elements of randomization.

CCKO was led by 3 pediatric tertiary care children’s hospitals in partnership with 9 affiliated community satellite clinics (eTable 1 in Supplement 2). The sites were chosen for their readiness to deliver coordinated care using the CCKO model and spread across Ontario (the catchment area of the participating sites encompassed two-thirds of Ontario’s population).

Patients new to complex care 16 years or younger who satisfied Ontario’s standard operational definition for CMC (fragility, chronicity, complexity, and technology dependence and/or users of high-intensity care)^34^ were eligible for this study. Written informed consent was obtained from all participating caregivers (ie, parent or guardian), and consent/assent was obtained from children who were able to provide it. Further specifics on the definitions of each of the eligibility criteria can be found in the study protocol.^31^ Enrollment occurred between December 7, 2016, and May 31, 2019; the study was conducted through June 2021.

Patients were excluded from the evaluation if they needed to be seen urgently, defined as children with high utilization of hospital-level care (≥3 hospitalizations, ≥2 intensive care unit [ICU] admissions, ≥30 days of total hospitalizations in the previous 3 months, excluding newborn admission), a tracheostomy and home ventilation, or a highly fragile medical status. Patients were also excluded if they were already being followed up by a complex care team, had a sibling followed up by a complex care team, or had a caregiver with inadequate language skills to comprehend the study surveys or if their primary caregiver was not expected to be involved in the child’s care over the entirety of the 2-year study period (eg, a foster parent).

### Intervention Group

Intensive care coordination was led by a nurse practitioner and pediatrician in a structured complex care clinic at participating sites and has been described elsewhere.^27,35,36,37^ Within CCKO, care coordination included direct provision of clinical care; coordination of clinical care, appointments, follow-up, and goal setting; and facilitation of communication among members of the multidisciplinary care team and families through the creation, maintenance, and use of a complex care plan. The care plan was co-created by the nurse practitioner with patients and their families to support collaboration and coordination between tertiary care, primary care, rehabilitation, and home and community care. The nurse practitioner was the first point of contact for CCKO families and was accessible during weekday and daytime hours. All patients continued to receive primary care (eg, immunization, sick visits) from their primary care clinicians.

### Waitlist Group

The waitlist group received standard care from primary and specialty care clinicians during the waitlist period. At the end of 12 months, all CMC randomized to the waitlist group received the CCKO intervention.

### Outcome Measures

All trial end points were chosen based on their importance to good outcome, according to family caregivers and clinicians, through an iterative consensus process.^38,39,40^ A schematic of outcomes is summarized in eFigure 1 in Supplement 2. The consensus process identified 3 types of service delivery outcomes as co-primary outcomes, which were measured using specific indicators from the Family Experiences With Coordination of Care (FECC) survey. These service delivery outcomes were (1) coordination of care among health care professionals (FECC 8a and 8b), (2) coordination of care between health care professionals and families (FECC 5), and (3) utility of care planning tools (FECC 16 and 17). The content validity of the FECC was obtained from families of CMC, with satisfactory psychometric performance of construct validity with other measures, and known reliability of parent-proxy reporting among diverse samples of patients requiring complex care.^41^ These 3 co-primary outcomes were each quantified with an ordinal scale where larger values indicated better experiences. Outcomes were measured at baseline and at 6, 12, and 24 months. Of note, by the time of the 24-month assessment, both groups were receiving the CCKO intervention.

Secondary outcomes were also selected by the consensus process^38,39^ and included the following: (1) service delivery (all nonprimary FECC indicators); (2) children’s outcomes of quality of life, emotional health, and physical pain, measured by parent-proxy, respectively, using subscales from the KIDSCREEN-52^39,42^ and a 10-cm linear visual analog scale^43,44^; and (3) parent (or caregiver) outcomes of perceived physical health, mental health, fatigue and sleep disturbance (measured by the Patient Reported Outcomes Measurement Information System^45,46^), and satisfaction with life (measured with the Satisfaction With Life Scale).^47,48^ These outcomes were reported at the same time intervals as the primary outcomes.

Health care system costs in the first and second year after randomization were based on health care service use (inpatient, intensive care, emergency department, primary care and specialty clinics, prescriptions, and home-health care). This information was encoded from administrative health data housed at ICES (formerly the Institute for Clinical Evaluative Sciences) and used to estimate system costs using established case costing methodology.^7^

### Sample Size

The sample size was based on co-primary FECC domain outcome measures using the following criteria: (1) a 2-sided test of the null hypothesis at the 5% level, (2) power of 80%, and (3) 10% of participants lost to follow-up. The study team conservatively projected the total sample size to be 140 participants (70 per group). The projected smallest clinically important difference of 0.5 of the within-patient standard deviation was recommended by the developer of the FECC as a moderate effect size.^41,49^

### Randomization and Masking

Patients were randomized to their assigned group with 1:1 allocation with random block sizes between 6 and 8 within each stratum (center). Blinding of patients and investigators was not feasible, but data analysts were blinded to enrollment group.

### Statistical Methods

All analyses were conducted according to a modified intention-to-treat principle, excluding only patients with no data postrandomization (baseline or otherwise). Comparisons between the 2 groups were made for the 3 co-primary outcomes at the end of year 1 (12 months) and additionally at the end of year 2 (24 months) using ordinal regression using a cumulative logit model including center as a random intercept and adjusting for baseline values. The Holm-Bonferroni method was adopted to adjust for false positives due to chance of 3 simultaneous outcomes, where the overall target type I error rate was .05.^50^

Secondary child and parent outcomes were compared at 12 and 24 months with linear regression including center as a random intercept, adjusting for baseline values, and also applying the Holm-Bonferroni method.^50^ Health care utilization and costs were compared using *t* tests for means, the Kruskal-Wallis tests for medians, and χ^2^ for proportions.

### Sensitivity Analyses

All missing primary and secondary outcome data were imputed using multiple imputation by chained equations incorporating baseline variables, group assignment, and the corresponding outcomes at baseline and 6 months.^51^ To address potential bias resulting from delays in initiation of complex care coordination after randomization, health care utilization and cost analyses were repeated using the initial complex care clinic visit date as the start of the intervention until 12 months from that date and additionally in the second year postrandomization using data from ICES.

To account for health care changes due to COVID-19 restrictions, which affected most health care delivery across Canada beginning March 13, 2020, an additional health care utilization and cost comparison analysis was conducted using data truncated to March 13, 2020. We also conducted analyses excluding patients who died before the end of the observation period, who could not provide complete outcome data.

## Results

### Participant Characteristics

**Figure 1.**
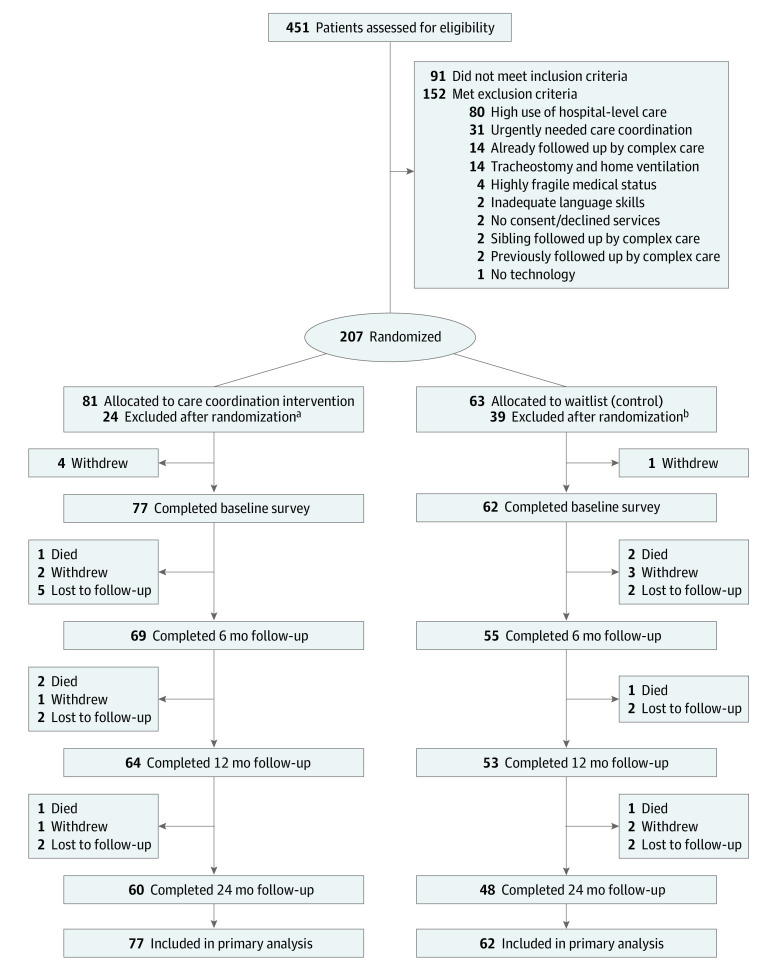
Trial Flow Diagram Among the 144 included included in the study, 3 participants did not have a valid identifier to link to administrative data. Therefore, 141 were included in the health care utilization and cost analysis (79 and 76 in the intervention group in year 1 and 2, respectively; 62 and 59 in the waitlist group in year 1 and 2, respectively). ^a^These participants were not included because the family declined services (n = 4) or did not give consent (n = 12), their language skills were inadequate to comprehend the study surveys (n = 5), or there was no continuous caregiver (n = 3). ^b^These participants were not included because they were already being followed up by a complex care team (n = 2), they died before consent was given (n = 2), the family declined services (n = 1) or did not give consent (n = 23), their language skills were inadequate (n = 6), or there was no continuous caregiver (n = 5).

A total of 451 participants were assessed for eligibility, of whom 207 fulfilled preestablished inclusion criteria for randomization and of whom 144 were eligible for inclusion in the study (Figure 1). Of these, 139 participants completed baseline surveys and composed the analytic sample for our primary analysis (77 in the intervention group and 62 in the waitlist [control] group), of whom 117 participants (84%) completed the 12-month follow-up and 108 (78%) completed the 24-month follow-up. Among the randomized patients, 141 had linked health administrative data available for health care cost analyses. The median age at enrollment for those included in the primary analysis was 29 months (IQR, 9-102); 83 participants (60%) were male. The mean (SD) number of diagnoses was 6.5 (3.3), medications was 5.6 (3.7), and technological devices was 2.9 (1.8).

The intervention and waitlist groups had similar baseline demographic and clinical characteristics (Table 1 for primary analysis and eTable 2 in Supplement 2 for health administrative data analysis). Participants were seen a median (IQR) of 88 days (54-119) and 364 days (348-382) postrandomization in the intervention and waitlist groups, respectively. Six patients died in year 1 (3 in each group) and 2 in year 2 (1 in each group), 11 patients withdrew in year 1 (7 in the intervention group and 4 in the waitlist group) and 3 in year 2 (1 in the intervention group and 2 in the waitlist group), and 10 were lost to follow-up in year 1 (7 in the intervention group and 3 in the waitlist group) and 4 in year 2 (2 in each group) (Figure 1 and eTable 3 in Supplement 2).

**Table 1.** Characteristics of Children and Parent Respondents at Baseline

Characteristic	No. (%)	
	Intervention group (n = 77)	Waitlist group (n = 62)
**Child demographic data**		
Time between study milestones, median (IQR), d		
Randomization to baseline survey completion	55 (30-90)	49.5 (34-79)
Baseline survey completion to first clinic visit	20 (0-61)	313 (281-336)^a^
Randomization to first clinic visit	88 (54-119)	364 (348-382)^b^
Age at enrollment, median (IQR), mo	29 (7-112)	33.5 (1-93)
Sex		
Male	45 (58)	38 (61)
Female	32 (42)	24 (39)
Self-reported ethnicity		
African	5 (7)	2 (3)
East Asian	11 (14)	6 (10)
Caribbean or West Indian	4 (5)	2 (3)
European	45 (58)	39 (63)
South Asian	6 (8)	6 (10)
Other^c^	6 (8)	7 (11)
Enrolled in school outside the home	27 (35)	23 (37)
**Clinical baseline characteristics**		
Children with hospital admissions between randomization and baseline completion	23 (30)	18 (29)
Primary diagnoses^d^		
Neurologic/neuromuscular	29 (38)	28 (45)
Cardiovascular	4 (5)	4 (6)
Respiratory	0	2 (3)
Kidney/urologic	4 (5)	1 (2)
Gastrointestinal	1 (1)	1 (2)
Hematologic/immunologic	0	1 (2)
Metabolic	2 (3)	0
Congenital/genetic defect	34 (44)	19 (31)
Premature/neonatal	2 (3)	2 (3)
No. of diagnoses, mean (SD)	7.0 (3.5)	6.0 (3.0)
No. of medications, mean (SD)	5.9 (4.1)	5.1 (3.2)
No. of technology devices used, mean (SD)	3.1 (1.9)	2.6 (1.7)
Technology device type		
Feeding^e^	57 (75)	49 (80)
Respiratory^f^	46 (61)	27 (44)
Mobility^g^	63 (83)	44 (72)
Other^h^	27 (36)	12 (20)
Hospital outpatient visits in previous year, mean (SD)^i^	14.2 (11.7)	11.3 (9.2)
Diet		
Oral	26 (34)	15 (24)
Enterostomy tube	45 (58)	39 (63)
Oral and enterostomy tube	6 (8)	8 (13)
Communication skills at age ≥12 mo^j^	56 (73)	48 (77)
Verbal	12 (21)	4 (8)
Nonverbal	37 (66)	35 (73)
**Parent (or caregiver) demographic data**		
Age, median (IQR), y^k^	33 (29-38)	34 (3-40)
Sex		
Male	14 (18)	7 (11)
Female	63 (82)	55 (89)
Marital status		
Married/common law	66 (86)	47 (76)
Single/widowed	8 (10)	9 (14)
Separated/divorced	3 (4)	6 (10)
Employment status		
Full-time	29 (38)	19 (31)
Part-time	16 (21)	4 (6)
Unemployed/homemaker	25 (32)	26 (42)
Receiving social assistance/disability/pension	6 (8)	8 (13)
Student	1 (1)	5 (8)
Highest education level		
Elementary school (some or completed)	1 (1)	0
Some secondary/high school	3 (4)	2 (3)
Completed secondary/high school	6 (8)	7 (11)
Some postsecondary	17 (22)	11 (18)
Received university or college degree	50 (65)	42 (68)

^a^ Two participants died and 1 withdrew from the study before the first clinic visit.

^b^ Two participants died and 1 withdrew from the study before the first clinic visit.

^c^ In the intervention group, other ethnicity groups reported were British, Iranian, Native, White/American, Mexican, and South American; in the waitlist group, other ethnicity groups were Filipino, Hispanic, Jewish, Middle Eastern, African Canadian, Ismaili Muslim, and Mixed.

^d^ Missing data for child primary diagnosis: 1 (1%) in the intervention group and 4 (6%) in the waitlist group.

^e^ Feeding devices include gastrostomy, gastrojejunal, and nasogastric tubes.

^f^ Respiratory devices include nebulizer, oxygen, noninvasive ventilation (continuous or bilevel positive airway pressure), tracheostomy, ventilation, and suction.

^g^ Mobility devices include wheelchair, special strollers, special seating, walker, stander, prosthetics, and ankle-foot orthoses.

^h^ Other devices include hearing aids, glasses, wheelchair van, mechanical lift, oxygen saturation monitors, catheters, scales, hospital bed, and communication devices.

^i^ Missing data for hospital outpatient visits: 18 (24%) in the intervention group and 17 (27%) in the waitlist group.

^j^ Missing data for communication skills: 7 (13%) in the intervention group and 9 (19%) in the waitlist group.

^k^ Missing data for parent age: 30 (39%) in the intervention group and 18 (30%) in the waitlist group.

### Outcomes

**Figure 2.**
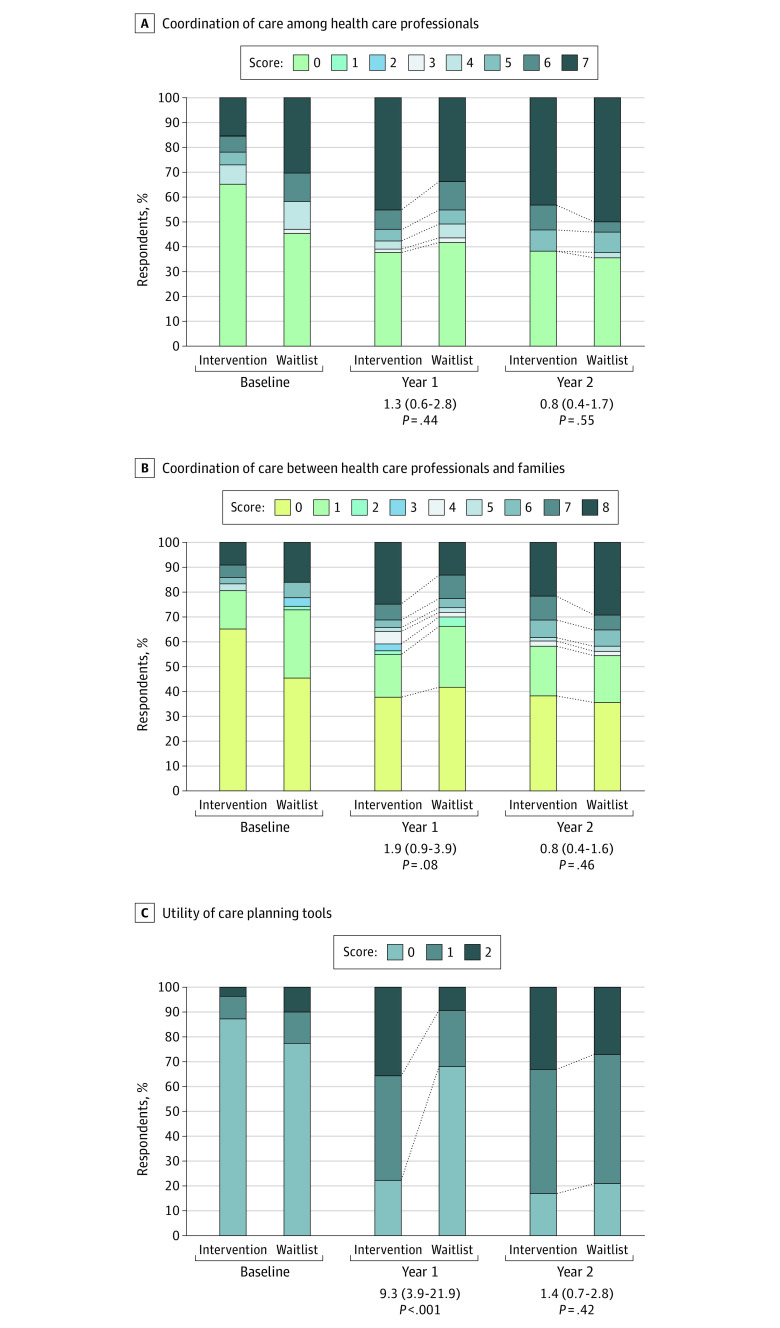
Comparison of Study Primary Outcomes Between Intervention and Waitlist Groups Family Experience With Care Coordination (FECC) scores were based on the sum scores of the indicators FECC 8a and 8b (coordination of care among health care professionals), FECC 5 (coordination of care between health care professionals and families), and FECC 16 and 17 (utility of care planning tools). Larger values indicate better perceived care. All *P* values are calculated based on comparisons of outcomes at the end of 12 months (year 1) and 24 months (year 2) using ordinal regression for FECC indicators. All models included center as a random intercept and are adjusted for baseline values and expressed as an adjusted odds ratio (95% CI), shown under each follow-up time with the *P* value. The comparison in panel C for year 1 reached statistical significance according to thresholds based on the Holm-Bonferroni method to adjust for false positives, where the overall target type I error rate was .05.

**Figure 3.**
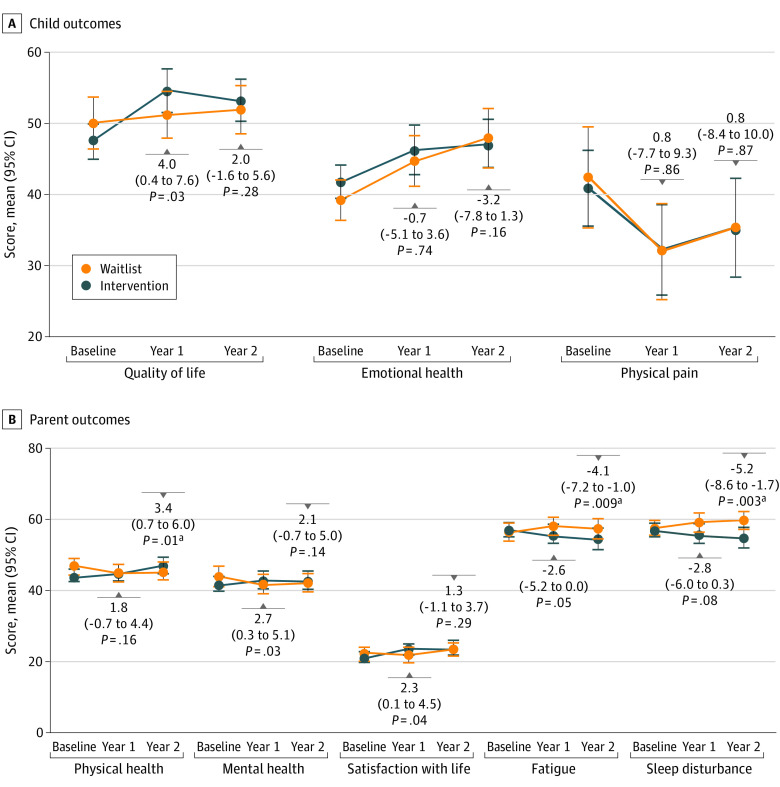
Comparison of Parent-Reported Study Secondary Outcomes Between Intervention and Waitlist Groups Child outcomes include quality of life; emotional health, measured by parent-proxy respectively using subscales from the KIDSCREEN-52^39,42^; and physical pain, measured with a 10-cm linear visual analog scale.^43,44^ Parent or caregiver outcomes include perceived physical health, mental health, fatigue and sleep disturbance (measured by the Patient Reported Outcomes Measurement Information System^45,46^), and satisfaction with life (measured with the Satisfaction With Life Scale).^47,48^ Higher scores indicate more of the outcome of interest, which can mean improvement (child quality of life and emotional health; parental physical health, mental health, and satisfaction with life) or deterioration (child physical pain, parent fatigue and sleep disturbance) depending on the outcome. All outcomes were modeled using linear regression including center as a random intercept, adjusted for baseline values, and expressed as an adjusted mean difference (95% CI), shown above or below each follow-up data point. ^a^Statistical significance using thresholds based on the Holm-Bonferroni method to adjust for false positives, where the overall target type I error rate was .05.

All primary and secondary caregiver reported outcomes are shown in Figure 2 and Figure 3 and eTable 4 in Supplement 2. At 12 months, the scores for utility of care planning tools were more positive in the intervention group compared with the waitlist group (adjusted odds ratio [aOR], 9.3; 95% CI, 3.9-21.9; *P* < .001; Holm-Bonferroni *P* value threshold for significance of .02), but there were no significant differences between the intervention and waitlist groups for coordination of care among health care professionals (aOR, 1.3; 95% CI, 0.6-2.8; *P* = .44) or coordination of care between health care professionals and families (aOR, 1.9; 95% CI, 0.9-3.9; *P* = .08). By 24 months of follow-up, there was no significant difference in any of the co-primary outcomes between the 2 groups. Five of 17 FECC indicators (FECC 6, 9, 10, 16, and 17) were significantly improved based on Holm-Bonferroni thresholds at 12 months, and none differed between groups at 24 months (eTable 5 in Supplement 2).

For both child and parent outcomes, no differences met Holm-Bonferroni thresholds for significance at either 12 or 24 months. By 24 months, significant improvement was reported in the intervention group for parent physical health (adjusted mean difference [aMD], 3.4; 95% CI, 0.7 to 6.0; *P* = .01), fatigue (aMD, −4.1; 95% CI, −7.2 to −1.0; *P* = .009), and sleep disturbance (aMD, −5.2; 95% CI, −8.6 to −1.7; *P* = .003).

Health care use was similar between the 2 groups in year 1 (Table 2). Health care costs were similar in year 1 between the intervention and waitlist groups (median, CAD$52 804; IQR, 20 520-108 608, vs CAD$50 176; IQR, 24 188-$120 122 [median, US $39 593; IQR, 15 386-81 435, vs US $37 622; IQR, 18 136-90 068]; *P* = .90). Slightly more participants in the intervention group used home care (94.9% vs 83.9%; absolute difference, 11.1%; 95% CI, 1.2-20.9; *P* = .03), but there were no differences in admissions, length of stay, ICU use, emergency department visits, outpatient visits, or primary care visits. Total year 2 health care costs were lower in the intervention group (median, CAD$17 891; IQR, 6098-61 346, vs CAD$37 524; IQR, 9338-119 547 [median, US $13 415; IQR, 4572-45 998, vs US $28 136; IQR, 7002-89 637]; *P* = .01). There were no significant differences between the 2 groups for any individual health care use category. Results for sensitivity analyses did not meaningfully change study findings (eFigures 2 and 3 and eTables 6-11 in Supplement 2).

**Table 2.** Health Care Service Cost and Utilization Among Study Participants in the Intervention and Waitlist Groups in Year 1 and Year 2

Variable	Year 1^a^				Year 2^b^			
	Intervention (n = 79), median (IQR)	Waitlist (n = 62), median (IQR)	MD (95% CI)^c^	*P* value	Intervention (n = 76), median (IQR)	Waitlist (n = 59), median (IQR)	MD (95% CI)^c^	*P* value
Health care service cost, CAD$	52 804 (20 520 to 108 608)	50 176 (24 188 to 120 122)	2628 (−28 821.95 to 31 431.95)	.90	17 891 (6098 to 61 346)	37 524 (9338 to 119 547)	−19 633 (−46 272.91 to 8126.91)	.01
Cost, US$	39 593 (15 386 to 81 435)	37 622 (18 136 to 90 068)	1971 (−21 610.93 to 23 567.92)		13 415 (4572 to 45 998)	28 136 (7002 to 89 637)	−14 721 (−34 695.79 to 6093.62 )	
**Health care service utilization**								
Admissions	2 (1 to 4)	2 (0 to 4)	0 (−0.9 to 0.9)	.82	1 (0 to 2)	1 (0 to 3)	0 (−0.9 to 0.9)	.73
Inpatient LOS	4 (0 to 18)	3 (0 to 16)	1 (−3.7 to 5.7)	.88	1 (0 to 5)	2 (0 to 9)	−1 (−3.4 to 1.4)	.24
ICU LOS^d^	2 (1 to 11)	3 (1 to 7)	−1 (−6.7 to 4.7)	.99	3 (1 to 5)	9 (3 to 10)	−6 (−11.2 to −0.8)	.10
Visits								
ED	2 (1 to 4)	2 (1 to 4)	0 (−0.9 to 0.9)	.64	1 (0 to 2)	1 (0 to 4)	0 (−0.9 to 0.9)	.27
Primary care	3 (1 to 6)	3 (1 to 7)	0 (−1.6 to 1.6)	.81	1 (0 to 3)	1 (0 to 3)	1 (−0.9 to 0.9)	.60
Specialist	15 (11 to 20)	13 (8 to 20)	2 (−1.2 to 5.2)	.24	12 (8 to 17)	12 (7 to 17)	−1 (−4.4 to 2.4)	.41
Prescriptions	27 (16 to 48)	30 (15 to 67)	−3 (−19.3 to 13.3)	.52	24 (5 to 42)	28 (11 to 69)	−4 (−21.0 to 13.0)	.20
Home care, No. (%)	75 (94.9)	52 (83.9)	11.1 (1.2 to 20.9)	.03	68 (89.5)	53 (89.8)	−0.4 (−10.8 to 10.1)	.95

Abbreviations: ED, emergency department; ICU, intensive care unit; LOS, length of stay; MD, median difference.

^a^ Total n = 141 among the 144 eligible as 3 participants did not have a valid identifier to link to administrative data.

^b^ Total n = 135 for year 2 analysis as 6 participants (3 in the intervention group and 3 in the waitlist group) died by the end of year 1.

^c^ MD calculated as the difference between intervention and waitlist groups. *P* values were calculated using Kruskal-Wallis tests for medians and χ^2^ for proportions.

^d^ ICU LOS calculated among those with an ICU stay. In year 1, 20 participants in the intervention group and 14 in the waitlist group had an ICU stay. In year 2, 11 participants in the intervention group and 12 in the waitlist group had an ICU stay.

## Discussion

We found improvement in perceived utility of care planning tools in those receiving the CCKO intervention compared with those waiting to receive it at 1 year, but no significant differences were observed in perceived care coordination among health care professionals or between clinicians and families. No significant differences were observed in a variety of child outcomes, parent (or caregiver) outcomes, and health care system utilization measures between the 2 groups at 1 year. When both groups were receiving the CCKO intervention at the end of the second year, parent physical health, fatigue, and sleep disturbance improved and overall year 2 costs were lower among those who had been randomized to receive the CCKO intervention earlier, exceeding in magnitude the estimated CAD$6500 (US $4874) annual cost per patient for implementing CCKO.

This study adds to the limited literature evaluating structured pediatric complex care programs using RCT designs and is the first trial including CMC outside of a single health care setting (eg, a children’s hospital). While there has been a rapid proliferation of such programs in the United States nationally and internationally,^37,52,53,54,55,56^ few studies have used controlled designs. A parallel-group RCT including 201 patients from a single center in Houston reported a reduction in rates of serious illness, including emergency department visits, hospitalizations, and ICU care, with comprehensive care in a medical home model at a tertiary care hospital.^23^ An additional RCT with 342 patients from the same center reported that adding a hospital consultation service reduced hospital days, hospitalizations, ICU days, and health care system costs,^25^ and a third RCT with 422 patients from this group of investigators found that the addition of telemedicine to complex care reduced days outside the home, serious illnesses, and health care costs.^24^ In contrast, a cluster RCT randomizing primary care clinicians to access to complex care services for their patients reported no change in child functional status or hospital-based utilization and increased overall costs.^26^ Differences in findings across studies can be attributed to characteristics of the intervention, implementation challenges when scaled, and/or different outcome measures.

In our study, we prioritized parent or caregiver and clinician perspectives for outcome measure choices,^40^ which included outcomes that may be less responsive to change from the CCKO intervention. For instance, the lack of improvement in parent reports of care coordination across clinicians may be attributable to the challenges of improving communication between subspecialists when the complex care clinic is not directly involved in these interactions. Health care utilization and quality of life may also be difficult to change because of the underlying medical complexity and fragility of the children enrolled. Further, the implementation of CCKO across 12 sites may have led to heterogeneity in the implementation of the CCKO model.^30,32^ Due to the pragmatic nature of the trial, there were delays for some patients in the intervention group being seen (the median date of the first appointment was 3 months postrandomization), which may explain why some differences between the groups may not have been apparent at 12 months. Improvements in cost and some other outcomes at 24 months may be due to a delay in outcome measure improvement with CMC care coordination or other factors, because both groups were receiving the intervention at that time.

### Strengths and Limitations

The study has limitations. To meet ethical standards, recruitment was limited to children who were not deemed to urgently need complex care. Many of these patients are those who are most likely to benefit from CCKO, as were those with other exclusion criteria (eg, language skills). Although we attempted to create a comprehensive outcome measurement framework, collection of self-reported data on children was limited to parental proxies because of the very high prevalence of intellectual disabilities in the population. We used an outcome measure validated specifically for CMC research (the FECC) that aligned with family-prioritized outcomes, but the measure has not been previously used for evaluative research designs. While we had near-complete data on health care use and cost, there was some loss to follow-up for parent-reported data. We may have been underpowered even with imputation to detect some potentially clinically important differences, or the waitlist period was too short to find differences in some secondary outcomes. Lastly, CCKO was conducted within a specific health care system and included a population definition that may differ from others (eg, inclusion of technology dependence and/or high-intensity care as a criterion). Generalization to other settings (eg, those without universal health care and/or different availability of home health service) may be limited.

## Conclusions

A waitlist randomized implementation of a pediatric complex care intervention that assigned a nurse practitioner–pediatrician dyad partnering with families to provide intensive care coordination and comprehensive plans of care led to better scores for utility of care planning tools compared with the waitlist group at 1 year but not for other care coordination, child outcomes, parent outcomes, and health care system utilization. Improvement in some outcomes at 2 years, including costs, suggests that extended evaluation periods may be helpful in assessing pediatric complex care interventions.

## Notes

Supplement 1. Trial protocol

Supplement 2. **eFigure 1**. Primary and Secondary Outcome Schematic **eFigure 2.** Comparison of study primary outcomes between intervention and waitlist groups after multiple imputation **eFigure 3.** Comparison of parent-reported study secondary outcomes between intervention and waitlist groups after multiple imputation **eTable 1.** CCKO regional and community complex care clinics and breakdown of participants from each site **eTable 2.** Baseline characteristics of participants with complete health administrative data **eTable 3.** Baseline characteristics of participants with and without loss to follow-up at 12 and 24 months **eTable 4.** Caregiver-reported secondary outcomes between the intervention and waitlist group at baseline, month 12 and month 24 follow-up **eTable 5.** Individual Family Experiences with Care Coordination (FECC) outcomes at baseline, month 12 and month 24 follow-up **eTable 6.** Caregiver-reported secondary outcomes between the intervention and waitlist group at baseline, month 12 and month 24 follow-up with imputed missing data (multiple imputation) **eTable 7.** Individual Family Experiences with Care Coordination (FECC) outcomes at baseline, month 12 and month 24 follow-up with imputed missing data (multiple imputation) **eTable 8.** Health service cost and utilization among study participants using the initial complex care clinic visit date as the start of the intervention **eTable 9.** Health service cost and utilization among study participants who had their entire Year 1 and Year 2 follow-up before estimated onset date of the pandemic (March 13, 2020) **eTable 10.** Health service cost and utilization among study participants, excluding deaths in Year 1 and Year 2 **eTable 11.** Health service cost and utilization among study participants who had their entire 1^st^ and 2^nd^ year follow-up before estimated onset date of the pandemic (March 13, 2020), excluding deaths in Year 1 and Year 2

Supplement 3. Data sharing statement

Supplement 4. Protocol changes
