# Supplementary material for: Effectiveness of Structured Care Coordination for Children With Medical Complexity: The Complex Care for Kids Ontario (CCKO) Randomized Clinical Trial
Source: JAMA Pediatr. 2023 Mar 20;177(5):461–71. doi: 10.1001/jamapediatrics.2023.0115 (PMC10028546; doi:10.1001/jamapediatrics.2023.0115)
Supplement: Supplement 1. — Trial protocol [file jamapediatr-e230115-s001.pdf]

Research Protocol

Complex Care for Kids Ontario (CCKO): A patient- and family-centred implementation and evaluation of care coordination for children with medical complexity

Table of Contents

1.0 Administrative Information ... 2
2.0 Introduction ... 3
2.1 Background and Rationale ... 3
2.2 Overarching Aim ... 4
2.3 Trial Design ... 4
3.0 Methods ... 6
3.1 Study Setting ... 6
3.2 Eligibility Criteria ... 6
3.3 Interventions ... 7
3.4 Outcomes ... 8
3.5 Participant Timeline ... 12
3.6 Sample Size ... 13
3.7 Recruitment ... 13
3.8 Randomization – Sequence Generation, Allocation Concealment and Implementation ... 14
3.9 Blinding ... 14
3.10 Data Collection Methods ... 14
3.11 Data Management ... 15
3.12 Statistical Methods and Data Analysis ... 15
3.13 Monitoring ... 18
4.0 Ethics and Dissemination ... 19
4.1 Research Ethics Approval ... 19
4.2 Confidentiality ... 19
4.3 Dissemination policy ... 19
References ... 20
Appendix 1 – Strategic Framework for CCKO ... 23
Appendix 2 – CCKO Planning and Implementation Regions ... 24

1.0 Administrative Information

Trial Registration: ClinicalTrials.Gov Identifier: NCT0298757

Funding: Canadian Institute of Health Research (CIHR), the Ontario Strategy for Patient Oriented Research, and the Provincial Council for Maternal and Child Health (PCMCH) in Ontario

Roles and Responsibilities:

Principal Investigator:
Eyal Cohen, Hospital for Sick Children, University of Toronto, Toronto, Ontario
Email: eyal.cohen@sickkids.ca Phone: 416-813-1500

Co-Principal Investigator: Astrid Guttmann, Hospital for Sick Children, University of Toronto, Toronto, Ontario

Knowledge User: Lisa Osqui, Provincial Council for Maternal and Child Health, Toronto, Ontario

Knowledge User: Sanober Diaz, Provincial Council for Maternal and Child Health, Toronto, Ontario

Ottawa Region Lead: Nathalie Major, Children`s Hospital of Eastern Ontario, Ottawa, Ontario

Greater Toronto Region Lead: Julia Orkin, Hospital for Sick Children, Toronto, Ontario

Clinical Trial Methodologist: Martin Offringa, Hospital for Sick Children, University of Toronto, Toronto, Ontario

Measurement Scientist: Nora Fayed, Queen`s University, Kingston, Ontario

Qualitative Research Expert: Niina Kolehmeinen, Newcastle University, Newcastle upon Tyne, United Kingdom

Health Economist: Myla Moretti, Hospital for Sick Children, Toronto, Ontario

Analyst: Abby Emdin, Hospital for Sick Children, Toronto, Ontario

Analyst: Longdi Fu, ICES, Toronto, Ontario

Epidemiologist: Sima Gandhi, ICES, Toronto, Ontario

Statistician: Andy Willan, SickKids Research Institute, University of Toronto, Toronto, Ontario and Anna Heath, SickKids Research Institute, University of Toronto, Toronto, ON

2.0 Introduction

2.1 Background and Rationale

Medical and surgical advances have led to falling mortality rates for almost every life-threatening condition of childhood, such as extreme prematurity (1), complex congenital anomalies (2), and congenital or acquired brain injury (3). Technologic advances such as ventilator support, feeding tubes and transplantation have successfully prolonged the lives of children and youth with lung, gut and other organ failure. This epidemiologic transition (4) has created a burgeoning population of children with medical complexity (CMC) – children with new morbidities, which are caused by longer survival itself as well as the complications of the therapies that have prolonged their life expectancy.

CMC are defined as "children with chronic conditions with elevated service needs, functional limitations and high healthcare use" (5). About 0.7% of children are CMC (41,500 in Canada), yet they use a remarkable one-third of all child health resources (~$1.1 billion/year in Canada) (6), accounting for 43% of child deaths, 49% of hospital days, and 75-92% of consumed assistive health technology (7, 8). CMC, their parental caregivers and families, and the healthcare system that support them endure enormous challenges, including: multiple and prolonged hospitalizations (9), frequent medical errors (10), poor care coordination (9, 11), and extraordinary stress on parental caregivers (12). The consequences include poor caregiver health (13), marital discord (14), and profound negative financial impact (15).

Our team has shown through a series of before- and after- studies, that targeted and integrated complex care interventions may improve the health outcomes of CMC (16), including reducing the extraordinary burden of caregiving on their families (17) and mitigating costly and unnecessary healthcare expenditures (18, 19). However, the validity of these findings and those of other investigators has been limited by small sample sizes, lack of control groups and incomplete outcome measures (20).

Within its mandate as a provincial program, and supported by Ontario's Ministry of Health and Long-Term Care (MOHLTC), the Provincial Council of Maternal and Child Health (PCMCH) has implemented the Complex Care Kids Ontario (CCKO) Strategy (**Appendix 1**). CCKO will help improve care for CMC who are Medically Fragile and/or Technology Dependent (MFTD) and their families by providing an integrated approach to medical care and coordination. The strategy also aims to improve service delivery, health, and quality of life of the patient-families involved. Over four years (2016-2020), ~6,200 CMC across five regions will receive tailored personalized care plans and coordination from a key worker to complement other integrated care initiatives such as Health Links (21). The five CCKO Regions (four serving the catchment area of Ontario's children's hospitals, and one dedicated to Northern Ontario) will be established beginning in July 2016 to plan and implement integrated, coordinated healthcare across Ontario (**Appendix 2**). Each region will be responsible for care coordination across acute care, primary care, rehabilitation, home and community care providers. This will be facilitated by dedicated key workers to establish seamless integrated care teams including innovations to: reduce unnecessary

uncoordinated face-to-face appointments with many subspecialists, and develop and maintain a single, comprehensive and collaborative care plan that is designed to meet the child's/family's goals and optimize health outcomes.

2.2 Overarching Aim

The goal of this project is to develop a robust evaluation of the province-wide complex care intervention, CCKO, and test whether dedicated key workers and coordinated patient-family care plans have an impact on improving service delivery and quality of life for CMC and their families. Our primary objective is to evaluate the rollout of CCKO using a randomized wait-list design and mixed-methods (qualitative and quantitative) research.

Primary Research Question:

1. a) What is the comparative effectiveness of the CCKO intervention relative to the waitlisted group?
   b) What are the elements that explain the successes and gaps of the intervention?

Hypothesis: We hypothesize CCKO will lead to improved variety of patient- and family-centred outcomes, while decreasing unnecessary healthcare utilization. In particular, we hypothesize that the intervention will:

i) Improve coordination among health care providers and families;
ii) Improve children's and parents' quality of life by decreasing the amount of time and effort spent on coordination of care;
iii) Decrease parental fatigue;
iv) Improve families' experiences with their child's health care;
v) Decrease utilization of health services outside the immediate scope of care (i.e., hospital admissions, ER) as a result of improved coordination.

2.3 Trial Design

CCKO will utilize a wait-list variation of a randomized controlled trial design (**Figure 1**). The wait-list approach involves rolling out an intervention over time, whereby all participants are randomized into two groups (A and B) to receive the intervention at different time points determined at random (22,23). Group A would receive the intervention immediately (when resources become available; at time 0). Group B would be on the wait-list and receive the intervention after 12 months. This study design uses the time period before the intervention as the control period/baseline, to be compared in parallel to those receiving the intervention (22). Baseline measurements would be collected at month 0, and Groups A and B would be compared at months 6 and 12.

Figure 1 – Trial Design

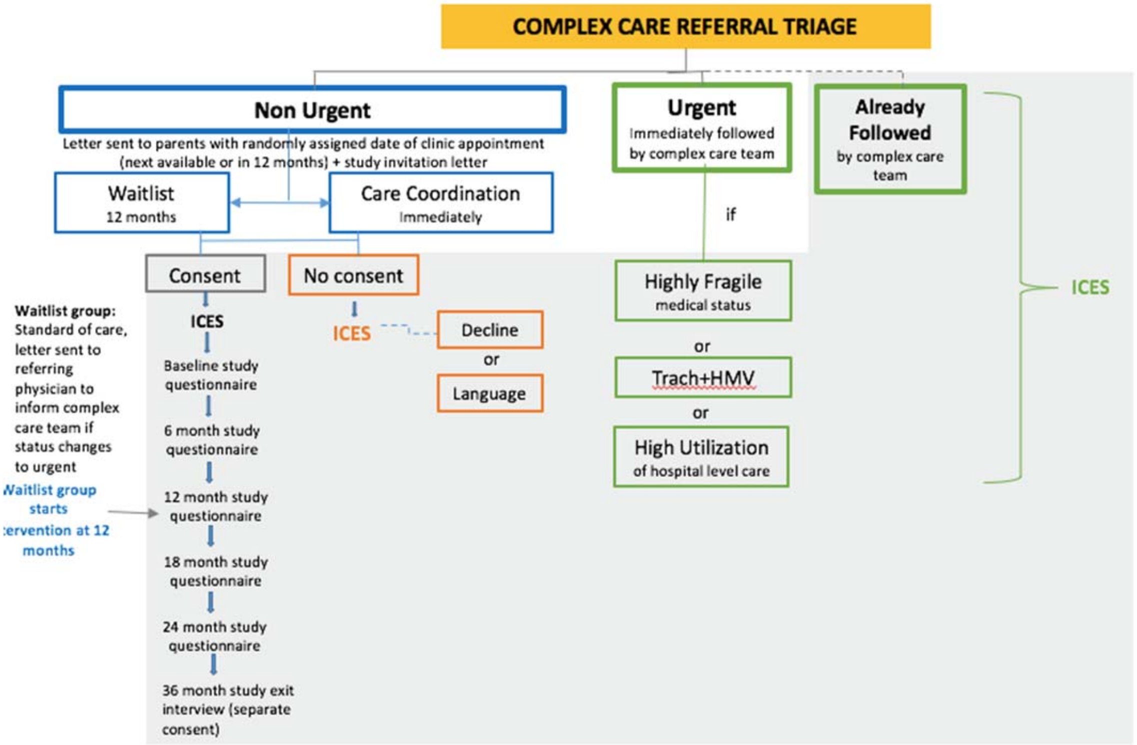

The wait-list design is used in scenarios where it is considered unethical to withhold an intervention or treatment with proven benefits, or if there are logistical or financial constraints that prevent the intervention from being administered at one time point (22,23). This design generates evidence of an intervention program's cost-effectiveness by leveraging real-world operational need for staggered rollout of CCKO to all eligible children for logistical reasons (e.g. high workload for key workers in the first few weeks to months after meeting families in order to create care plans and foster relationships). All CCKO sites intend to deliver care coordination to all eligible children. However given human resource limitations, CCKO program implementation will require the creation of a waitlist in order to be able to include all the patients. With the exception of patients for whom care coordination is urgently required (see exclusion criteria a)-c) in 3.2), all patients referred to CCKO will randomly assigned to either receive the intervention immediately (at the soonest available clinic appointment) or after the waitlist period (1 year). From a research perspective, this approach minimizes the risk of selection bias by retaining the design element of randomization.

3.0 Methods

3.1 Study Setting

The CCKO evaluation will take place at three pediatric tertiary care children`s hospitals (The Hospital for Sick Children [SickKids] Toronto, ON, Children`s Hospital of Eastern Ontario [CHEO], Ottawa, ON; Hamilton Health Sciences, Hamilton ON), and their respective satellite clinics (Michael Garron Hospital, Toronto, ON, North York General Hospital, Toronto, ON, Credit Valley Hospital, Mississauga, ON, Peterborough Regional Health Centre, Peterborough, ON, Royal Victoria Hospital, Barrie, ON, Orillia Soldiers' Memorial Hospital, Orillia, ON). These sites were chosen due to their size, relative proximity, spread across Ontario, as well as interest in collaborative work to improve the health of CMC. An additional tertiary care children's hospital, London Health Sciences Centre, will be involved in CCKO, but not in this evaluation. Further sites in Northern Ontario are also anticipated to join CCKO, but will also not be part of this evaluation.

3.2 Eligibility Criteria

The target study population includes children who satisfy the Standard Operational definition for CMC who are MFTD living in the catchment area of participating centres. The specific inclusion criteria are summarized in **Figure 2**. Patients will be identified from either practices and/or health care organizations.

Exclusion Criteria:

a) High Utilization of hospital level care
    - ≥ 3 hospitalizations, ≥ 2 ICU admissions, ≥ 30 days of total hospitalization in previous 3 months, excluding newborn admission
b) Patient with tracheostomy and home ventilation
c) Medical Status is deemed highly fragile and the need for close follow-up is deemed essential by both referring and triaging team
d) Already followed by a complex care team
e) >16.0 years of age
f) Inadequate English language skills to comprehend study questionnaires
g) Parent will not be involved in child's care over entirety of study period (2 years)

Patients whose caregivers cannot complete questionnaires in English will not be involved in the primary analysis which involves patient-reported outcome measures (PROMs) and patient-reported experience measures (PREMs) that are written and validated in English, but in order to ensure equitable access to this novel service, they will be allowed to enrol in the clinic and if possible provide health care utilization data via health card linkages at ICES that will be used in secondary analyses. Similarly, we will exclude those patients who are thought to be in need of urgent care coordination by their clinicians (e.g. acuity deemed urgent for care coordination due to new technology such a tracheostomy/ventilation or significant medical complexity with an prolonged inpatient stay), but these patients will contribute data with consent through linkage through their health card to routinely available health administrative data.

Figure 2: Definition of CMC who are MFTD

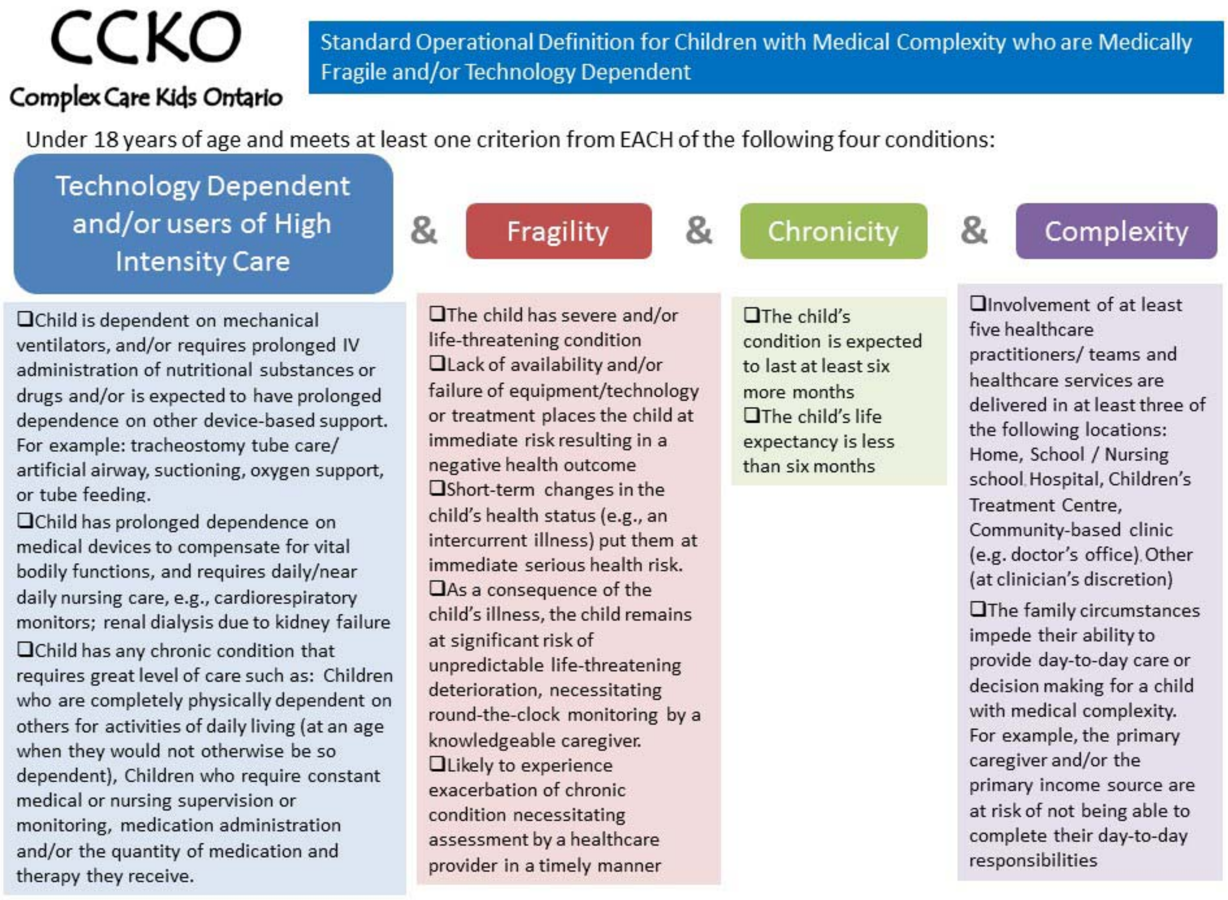

3.3 Interventions

Patients will be randomized to receive either *usual care* for 1 year prior to their first clinic appointment (wait list group) or enrolled to be seen as soon as possible in a *complex care clinic* as part of the CCKO initiative. All eligible patients will be randomly assigned to wait list or the clinic prior to being approached for consent to participate in the evaluation of CCKO. The CCKO intervention involves intensive care coordination, defined as: "deliberate organization of patient care activities between two or more participants (including the patient) involved in a patient's care to facilitate the appropriate delivery of health care services. Organizing care involves the marshaling of personnel and other resources needed to carry out all required patient care activities and is often managed by the exchange of information among participants responsible for different aspects of care" (24). Within CCKO, intensive care coordination will specifically include: 1) the tailored, family/health care provider co-creation and regular updating of care coordination plans for each child which will be 2) facilitated and accounted for by key

workers partnering with families. The key worker would have a nursing or nurse practitioner background and will support providers in enacting the coordinated care plan between acute care, primary care, rehabilitation, home and community care. The key worker would be available to provide advice from Monday to Friday, 9 AM – 5 PM, and will also develop plans of care for emergencies after hours as part of care plan development. Resources to maintain intervention fidelity among key workers will be created and checked with oversight by PCMCH and the workers will connect annually in a meeting that will be co-attended by family representatives.

The wait list group consists of CMC who are receiving various types and levels of care from primary and specialty care providers, and are waitlisted for complex care clinic (pre-enrolment control period). Standard of care during the control period will involve care delivered through a primary care provider (family physician or paediatrician), with subspecialty consultation as needed. This is the model of care for the vast majority of CMC in Ontario; among ~6,200 CMC in Ontario, only ~500 receive care in a structured complex care clinic. At the end of 1 year, all CMC randomized to the wait list group will be offered enrolment in the complex care clinic and data will continue to be collected for 1 additional year on all participants in an extension phase.

3.4 Outcomes

In partnership with health care providers and CMC families, a Family-Engagement Evaluation was conducted to identify patient-centered criteria for determining effectiveness of the intervention. The core set of relevant PROMs and PREMs was created in a consensus meeting using data collected from an online survey completed by 48 families of children aged 2-21 years from all five CCKO regions, as well as 86 health care providers. This core set informed the development of a framework for CMC as summarized in **Figure 3**.

Figure 3 – The CMC Family-Engagement Evaluation Framework

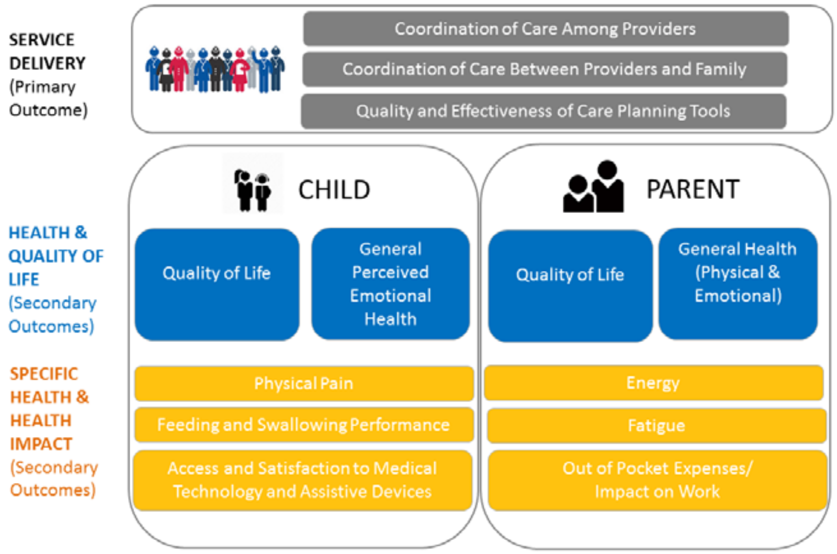

Measures representing these outcomes are selected based on their content applicability to the outcomes, proven psychometric performance (reliability and validity) among children and families with chronic health conditions, and the option for child report wherever possible. The family-Engagement Evaluation

includes general and specific outcomes within the domains of service delivery (primary and secondary outcomes); child outcomes (secondary) and parent outcomes (secondary). Feeding and swallowing performance, as well as satisfaction with medical devices and assistive technology, were identified as priorities for the evaluation by patients and clinicians, however these PROMs could not be found in the literature. As part of the Family-Engagement Evaluation, a team of complex care healthcare providers and measurement specialists will be working on developing appropriate measures for these core outcomes. However for the purpose of this study, only outcomes for which there are validated tools will be measured.

An overview of the included outcomes and their measures are presented in **Figure 4**. Selected outcomes will be assessed at enrolment (t=0), 6 months, 12 months, and 24 months post-randomization so that all subjects will be asked to provide two full years of data. A demographics survey will also be administered during enrolment to capture descriptive, background information of the two groups at baseline. Demographics information includes: sex, age, ethnic background, marital status, education level, and employment status. Qualitative interviews will be conducted at the end of the intervention with a subsample (n = 25) of families in order to capture their overall experience with the intervention, areas of improvement, barriers and/or challenges associated with the intervention, as well as perceived benefits.

Figure 4 – Overview of Outcomes and Associated Measures

Service Delivery Outcomes (Measurement Tool)	Parent Outcomes (Measurement Tool)	Child Outcomes (Measurement Tool)	Health System Outcomes (Measurement Tool)	Process Outcomes (Measurement Tool)
Coordination Among Providers (FECC^)	Life Satisfaction (KIDSCREEN, SWLS)	Life Satisfaction (KIDSCREEN)	Health Utilization (ICES) e.g. Hospital admissions, ER visits, etc.	Patient and family experience (Qualitative Interviews) e.g. Overall experience with intervention, barriers and facilitators, etc.
Coordination Between Providers and Families (FECC)	Overall Health (PROMIS)	Physical Pain (VAS)		
Utility of Planning/Follow-Up Tools (FECC)	Energy and Fatigue (PROMIS)	*Feeding and Swallowing Performance		
Individual family experiences with	Out of Pocket Expenses (Expense	*Satisfaction with Medical and		

care coordination indicators (FECC)	Diary)	Assistive Devices		

*Outcome measures to be developed as part of the Family-Engagement Evaluation; ^Primary outcome measure

Service Delivery (Primary) Outcomes: The service delivery outcomes identified as the most important from the consensus project were:

1. *Coordination of Care Among Health Providers and Families*
2. *Coordination of Care Between Health Providers and Families*
3. *Utility of Follow-Up Planning Tools*

These outcomes will be assessed with the Family Experiences with Coordination of Care (FECC) survey, as the primary outcomes measure for this study. The FECC has been validated on 1209 US CMC patient-families, and has been reported to have internal consistency >0.7 with proven discriminant validity for patient-family socioeconomic status and rurality (25), as well as responsiveness to change demonstrated in a recent CMC randomized control trial (RCT) (26). In the US sample on which it was validated, the FECC is composed of 20 separate indicators. Indicators within the FECC that map onto the family-provider prioritized outcomes from Ontario (Coordination of Care Among Providers and Families (FECC-8a, 8b); Coordination of Care Between Providers and Families (FECC-5); and, Care Planning Tools (FECC-16, 17) will be used to assess the primary study outcome.

3 of the 20 indicators will not be collected (FECC-15, 19, and 20) as they have content relating to: electronic-health records (FECC-19 and 20), which are not universally available in the region of the CCKO intervention; and translation services (FECC-15), which are less relevant in a study population limited to English-speakers. Individual FECC indicators will be assessed as secondary outcomes.

Child (Secondary) Outcomes: The child-specific outcomes that will be measured are:

4. *Quality of Life and Overall Emotional Health*
5. *Child's Physical Pain*

Children's quality of life and emotional wellbeing will be measured using the World Health Organization definition focused on subjective life appraisal (27) and a positive orientation of mood assessment respectively. These outcomes will be assessed using the using the "Feelings" subscale from the KIDSCREEN-52 (6 items), used in over 250 studies in the child health services literature since its publication in 2005 (28, 29); and represents the most suitable content overlap with CMC children.

Children's physical pain will be measured using only self or proxy reports of pain according to a 10 cm linear Visual Analog Scale (VAS) (30)**.** Linear VAS is considered to superior to other pain reports available for children due to consistencies of interpretation within parent-child dyads, test-retest reliability and measurement precision (31), and is most appropriate for the diverse functional ability of the CMC population.

Parent (Secondary) Outcomes: The parent outcomes identified as the most important from the consensus project were:

6. *Parents' Quality of Life*
7. *Perceived Emotional and Physical Health*
8. *Energy and Fatigue*
9. *Effects of Child's Condition on Parents' Finances and Ability to Work*

Parents' quality of life will be measured according to a subjective life appraisal definition with two scales.

The first will be Diener's highly validated Satisfaction with Life Scale (SWLS) (5 items) which is the most validated life satisfaction scale in health and social sciences literature (32, 33). The second is an adapted version of the KIDSCREEN survey subscale for Feelings (28,29).

Parents' perceived health, energy, and fatigue will be assessed with short forms of the Patient Reported Outcomes Measurement Information System (PROMIS) General Health (10 items); Sleep (8 items); and Fatigue (8 items) scales. These scales have been validated, have norm references data for comparison and have shown good to excellent psychometric properties among caregivers (34, 35).

Financial Impact on Parents' will be measured using an Expense Diary survey created by the co-investigators. This survey will capture financial impact based on lost time and ability to work, as well as out of pocket expenses for health care services, equipment, and travel using scales customized for CMC and standardized relative to various child health studies with the support of OCHSU investigators.

Health System Outcomes:
A cost-comparison will be presented as part of the trial findings. A cost-effectiveness analysis will be performed alongside this clinical trial to estimate the incremental costs (or savings) of the CCKO intervention compared to standard care in reducing hospitalizations. Both a health care system and societal perspective will be used with a time horizon of 12 months. Cost-effectiveness will be expressed as an incremental cost-effectiveness ratio (ICER) for each of the health care system and societal perspectives, expressed as point estimates and 95% confidence intervals. Given potential challenges in caregiver recall of out-of-pocket expenses and the lack of robust data on loss of productivity, the health care system perspective will be the primary health economic analysis.

Process Outcomes: Exit interviews with parents of children who received the intervention will be performed with the goals of: i) assessing the active ingredients of the intervention; ii) determining the fidelity of the intervention, iii) the 'dose' of intervention received, and iv) the elements of context that facilitate or hinder the intervention. All of these objectives will be probed with the purpose of learning the patient perspective of the intervention. Interviews as opposed to focus groups were chosen to encourage the unique experiences of each patient-family to come forward.

Detecting Response Shift: Parents and children affected by medical complexity have been shown to undergo a chronic disease adaptation process over time (36, 37). This can be a negative or positive shift in their expectations or priorities, which also effects their scoring of health and quality of life components. For example, if a parent expects a disease cure at baseline but shifts to an expectation that their child's

disease is managed to minimize morbidity after 12 months; a positive response shift has occurred. This is an adaptive phenomenon from a clinical perspective but can result in falsely concluding there was an improvement where there was none (Type I error). The error manifests an improvement in the response option scoring because the parent's calibration of poor to excellent on a Likert type scale shifted during the trial.

The opposite, negative response shift can also occur; the threshold for satisfaction with service provision can increase as a parent becomes more experienced. This can result in a negative change score even where there were authentic gains due to the intervention (Type II error). Mitigating this phenomenon called 'response shift' has a simple but rigorous solution called a 'then test' (38), which will be instituted for exploratory analyses. The then test will be implemented for the child (via the KIDSCREEN survey) and parent quality of life measures (using PROMIS tools) at 6, 12, 18 and 24 months for both intervention and waitlisted groups.

3.5 Participant Timeline

The CCKO Evaluation study timeline spans two years from 2016 to 2018, and will be nested in the CCKO Implementation Strategy running until 2020. The specifics of the timeline and milestones for this evaluation project are found in **Figure 5**.

Figure 5 – Study Timeline

	Allocation and Enrolment	Post-Allocation				Closeout
**Time point**	**-12 mo to 0 ($-t_1$)**	**0**	**6 mo (t_1)**	**12 mo (t_2)**	**24 mo (t_3)**	**36mo (t_4)**
Enrolment	X					
Eligibility screen	X					
Consent	X	X				
Allocation	X					
Assessments:						
Baseline data collection (i.e., demographics survey)		X				
Follow-up data collection		X	X	X	X	
Health care utilization				X	X	
Secondary analysis with ICES data				●——	——	——●
Data Analysis and Knowledge Translation			I	I	●——	——●

3.6 *Sample Size*

For the RCT, we conservatively project the total sample size to be 140 (70/arm) based on the following criteria: i) Two-sided test of the null hypothesis at the 5% level; ii) Power of 80%; iii) 10% lost-to-follow-up; projected smallest clinically important difference of 0.5 of the within-patient standard deviation, which is recommended by the developer as a moderate effect size (39).

The required sample size is considered feasible as it is estimated that a pool of about 250 patients are readily identifiable for recruitment at CCKO sites.

3.7 *Recruitment*

At the Complex Care Clinic at each site, staff that have legitimate access to their health and contact information will identify and provide a study information letter to eligible patients using the CMC definition in **Figure 2,** who will further be contacted by the research assistant. An approximate 50% recruitment rate from a pool of N=400 (200/year) is conservatively estimated based on previous recruitment and current waitlists. Families who are inpatient or who are attending a clinic visit will be approached in person for consent. Families may also be sent an information package consisting of consent

and assent forms to participate. The research assistant will follow up if a response is not received, and only families that agree to participate would be contacted to set up a phone or in-person interview (based on the method most convenient for participants). Given the geographical distance, it is not feasible to conduct all of the recruitment in person. Every effort will be made to conduct recruitment in person at the Complex Care Clinic, however some recruitment will need to take place over the phone. In order to document consent instances over the telephone, there is a telephone consent form. The questionnaires would take place at baseline, 6 months, 12 months, and 24 months. Upon completion of each set of questionnaires families will be provided with a $20 gift card to either Shoppers Drug Mart or Wal-Mart as a token of our appreciation for their time. Recruitment for the qualitative interviews would take place toward the end of the study, and a subsample of 25 families would be approached to answer questions about their overall experience with the Complex Care intervention.

3.8 Randomization – Sequence Generation, Allocation Concealment and Implementation

CCKO randomization will done using a computer-generated algorithm. Eligible children will receive the intervention in either Time 1 (approximately Jan 2017) or Time 2 (approximately Jan 2018). Randomization will be stratified by region/centre. Blocking will be used to ensure that the two comparison groups are about the same size throughout the trial for each site as well as for the trial as a whole. An allocation ratio of 1:1 with random block sizes between 6 and 8 will be used within each stratum (centre). This will help to ensure that clinicians or investigators will not decipher the block size. After patient consent is obtained, the study investigator or his/her delegate will assign the patient a unique identification (ID) number in sequential order that will be used throughout the study.

3.9 Blinding

Blinding of patients and investigators is not feasible. Data analysts will be blinded to the enrolment period.

3.10 Data Collection Methods

Upon obtaining consent, the Research Assistant will assign a research subject ID number to patients. Data collection for outcome measures will be mainly collected in hospital by the Research Coordinator (at SickKids) and Research Assistant at the other sites. Study PROMs have been carefully selected and prioritized, hence they are sufficiently brief to be administered at baseline, 6 months, 12 months, and 24 months.

Data collection will occur using iPads or hardcopy during visits in the waiting room before or after appointments. At home, patient-families can also fill in the surveys via the internet (RedCap) with a protected ID provided to them by the study staff or through an electronic copy that will be sent back to the research assistant. Online data collection methods have been articulated as more client-centered to the needs of patient families; their consent to participate is often conditional on this provision. Flexible methods of data collection will maximize diversity of respondents and maintain the involvement of vulnerable groups (such as low transportation access) in the trial.

Qualitative interviews will take place in person or over the telephone to maximize convenience for participants. Audio data from patient interviews will be recorded, transcribed verbatim and secured for anonymity.

3.11 Data Management

Data collection will be conducted by dedicated Research Assistants at each site, and synthesized by the Research Coordinator at SickKids. A study database will be designed and maintained as a secure Research Electronic Data Capture [REDCap] database. All data collection forms will be uploaded onto REDCap. The data on the various forms will be linked by a unique research subject ID number. The Research Coordinator will extract study data from questionnaires via the REDCap interface to complete the study-specific data collection forms on REDCap. An external user interface will also be created on REDCap for parents who opt to complete the surveys online.

All personal identifying information will be removed from the electronic study database. A separate secure list of research subjects' names and contact information will be maintained in an excel file for the purpose of the follow-up telephone interviews. All study-related electronic data files will be password-protected and reside on the Hospital server. Only members of the research team will have access to the server study file location via password-protected SickKids computers.

De-identified, password-protected databases with questionnaire data will be transferred to ICES through a Virtual Private Network from Sickkids. However, each individual site in partnership with their satellite clinics (Hamilton Health Sciences & CHEO) will be responsible for sending OHIP numbers to ICES. ICES will develop a data-sharing agreement with each site prior to the data exchange; the "data covenantor"- an ICES designate with special authority who is named at the Office of the Information Privacy Commissioner in Ontario to perform these duties - will act as the recipient. Sites will have the option to explore other data transfer modalities including web-based collection or an encrypted transfer with identification keys separated and transferred separately.

Further anonymization of transcripts will change names and other indirectly identifying information of participants as appropriate. The information the interview participants provide will not be linked or connected to the PROM data or ICES health utilization data in any way.

3.12 Statistical Methods and Data Analysis

<u>*Primary Analyses*</u>

i) Service Delivery (Primary) Outcomes: The primary analysis will be a comparison of the prioritized service delivery outcomes (Coordination of Care Among Health Providers and Families, Coordination of Care Between Health Providers and Families, Utility of Follow-Up Planning Tools) as measured with the FECC between the intervention and wait-list groups at the end of Year 1 (Month 12).

ii) Scoring Requisites: The Coordination of Care outcomes (among providers and between providers and families) are 'dependency' questions that need to be endorsed in order for the outcome to be assigned a score >0. Where a prior question limits a following question from being visible, the answer to the following question will be

considered the lowest level of care (0). For example, if the parent answers no to "Did the main provider/team help you to manage your child's care or treatment from these different doctors or care providers?" (Q3a), the answer to the following question "In the last 6 months, did the main provider/team seem informed and up-to-date about the care your child got from other providers?" (Q5a) was considered 'No' (0).

iii) Scoring: **Coordination of Care Among Health Providers** is based on the following questions in the FECC:Q5a, Q5b, and Q5d in FECC-8, with 3 possible responses that will be coded on an ordinal scale from 0 to 2, all of which are dependent on Q3a, response of '1' indicating patient's perception they have a care coordinator. All four questions will be added together to form an ordinal scale from 0 to 7 where larger values indicate better care. **Coordination of Care between Health Providers and Families** has four questions, Q9 and Q10 in FECC-5with 4 possible responses that will be coded on a 0-3 ordinal scale, which are dependent upon Q3a (patient perception they have a care coordinator=1) and Q7 (care coordinator has initiated communication=1). All four questions will be added together to form an ordinal scale from 0 to 8, where larger values indicate better care. **Utility of Follow-Up Planning Tools** will be based on 2 questions on shared care plans (Q29 in FECC-16 and Q32 in FECC-17) with dichotomous responses (0 or 1) that added together, will create an ordinal scale from 0 to 2, where larger values indicate better care.

These scores will be used as three co-primary outcomes. The appropriate descriptive statistics will be used to compare groups with respect to baseline and demographic variables. Each outcome variable at Month 12 will be compared between groups using an ordinal regression incorporating center as a random intercept and adjusting for the baseline score. A two-sided test of hypothesis will be applied. The Holm-Bonferroni method will be used to deal with multiple testing (the three outcomes), where the overall target type I error rate is 0.05. The principle of intention-to-treat will be applied to the final analysis.

iv) Child and Parent (Secondary) Outcomes: The data collection at 0 months and 6 months will be used to perform test-retest reliability, to establish baseline reference measures (0, 6, 12 months for this and other phases of intervention), and to assess for stability of outcome changes (24 months). Secondary child and parent outcomes will be compared at 0, 6 and 12 months using Bonferroni corrections to account for multiple testing. All secondary health system outcomes will be compared between the intervention and control as well.

v) Process Outcomes: Process data collected through patient interviews will be first analyzed one participant at a time without making distinctions regarding the specific sample criteria articulated above. Following that, distinct issues brought forward for the specific sample criteria will be compared and contrasted to the main sample results. All interview data will be analyzed using a descriptive approach. The overarching themes will be selectively coded using the categories of 'Active Ingredients, 'Fidelity', 'Dose', and 'Context'. The specific sub-themes under these pre-selected themes will be coded inductively.

vi) Health System Outcomes: A cost comparison analysis will be performed as part of the primary analysis. Additionally, cost-effectiveness will be subsequently performed, expressed as an incremental cost-effectiveness ratio (ICER), calculated by dividing the incremental costs of the intervention by the incremental difference in hospitalization days during the study period as the primary analysis and an ICER denominated on emergency department visits as a secondary analysis. Direct health care costs will include cost of the CCKO intervention and health services use by participants during the 12 month follow up period. Health services use by participants and covered by the provincial payer will be obtained through administrative data from the linkage with ICES. This will be expressed as absolute difference between the two groups with a 95% confidence interval. An interview administered expense diary will capture indirect costs, parent out-of-pocket expenses and additional health services covered by third-party payers. Intervention costs will include all Health Human Resources required to execute the intervention and will be allocated evenly across all participants. Extensive sensitivity analysis will be used to test the robustness of the results and explore uncertainty in any of the underlying assumptions. Probabilistic sensitivity analyses (PSAs) will also be used to establish the point estimate and 95% confidence interval for the ICERs obtained. The time horizon for the primary analysis will be one year, bounded by the date of randomization and the one year follow up interview.

<u>*Additional Analyses:*</u>

i) All analyses will be extended to 24 months to assess for any outcomes that may have delays in change and to assess for ongoing effects in enrolled subjects. All primary and secondary analyses will be conducted for Year 2 of the study comparing outcomes among patients in the two groups that they were assigned to.

ii) Comparison of PROMs and Interview Data: Qualitative interview data will be compared with PROMs to ascertain the association between experience-related themes and specific health outcome measures. Data on PROMs will be stratified based on scores for different outcomes, and compared with the qualitative data to gain a better understanding of how families' experiences with the intervention mapped on to patients' health-related outcomes.

iii) Sensitivity analysis #1: a) In order to address potential bias resulting from delays in initiation of complex care coordination after randomization, the primary analysis will be repeated using the initial complex care clinic visit date as the start of the intervention until 12 months from that date. b) To further explore this issue, an additional analysis will be performed through observation of the second year of outcome data post-randomization, using data from ICES. The comparison of Year 1 and Year 2 data will explore differences that may exist (in terms of group differences) and is expected to signal the importance of sensitivity analysis #1.

iv) Sensitivity analysis #2: To account for health systems changes due to COVID-19 restrictions, which impacted most health service delivery across the country beginning March 13, 2020, an additional cost-effectiveness analysis will be conducted. For this analysis all health

service data collection will be truncated as of March 13, 2020 and the ICER will be calculated including health services use up to that date.

v) Sensitivity analysis #3: Since health care utilization and costing data are being presented as per patient per year, in order to account for the small number of anticipated patients who may die or lose health insurance (OHIP) eligibility during the study, we will rerun the analyses excluding these patients. If this substantially changes study findings, data will be presented as per patient per month.

vi) Health Services Use Among Patients excluded from RCT: A secondary analysis will be performed after completion of the primary trial analysis for children who were excluded from randomization (e.g. those judged to urgently need care coordination), as well as those who cannot complete questionnaires (non-English speaking patients). For such children, we will try to find age- and sex-matched controls in the catchment area of London Health Sciences Centre (where there is no complex care program) with similar patterns of health utilization in the year prior to enrolment and meeting criteria for CMC using an administrative database definition that we have used previously. We will employ a differences-in-differences approach comparing changes in health services utilization and cost, patient- (or caregiver-) reported health-related quality of life, and family functioning, for all children before- and after- intervention, controlling for time trends. Other analyses will also determine which predictor variables distinguish trajectories for at risk groups for future work.

vii) In addition to a complete case analysis, since it was noted on original analysis that there was substantial missing data, the data were analyzed imputing missing values. Missing primary and secondary outcome data were imputed using multiple imputation by chained equations incorporating baseline variables, group assignment and the corresponding outcomes at baseline and 6 months.

3.13 Monitoring

Data Monitoring and Safety (DSMC) and interim analysis:
A DSMC does not seem necessary as there are no anticipated risks from participating. All wait-listed patients will be receiving standard of care, and there are no anticipated adverse events expected from a care coordination intervention. However, to detect early superiority, futility, or inferiority of the intervention, interim analyses are planned using the outcome data collected at 6 and 12 months. The trial PI and Steering group will be informed if a pre-defined change in the frequency of key patient and family outcomes is observed, whether positive or negative.

Adverse Event Reporting: While there are no adverse events expected in the context of the intervention being administered, any adverse events will be reported to the Research Ethics Board. All adverse events and adverse reactions will also be reported to the PI within 24 hours.

4.0 Ethics and Dissemination

4.1 Research Ethics Approval

The protocol will be reviewed by Research Ethics Boards at all participating sites. Study participants will be asked to provide written informed consent which will be obtained by the study Research Coordinator at SickKids and Research Assistants at other sites. The study Research Coordinator or Research Assistant will obtain informed consent from parental caregivers. Informed consent/assent will be obtained from all those who are able to provide it. Patients who decline to participate in the collection of PROMs and PREMs will be offered to consent for use of data on health services use from readily available administrative data. Health service data will also be collected on those who are excluded from participating (e.g. due to linguistic barriers or over 16 years old) by waiver of consent.

4.2 Confidentiality

All study data will be kept confidential by removing personal identifying information, and all study files will be maintained on a secure server with password protection. Anonymity will be maintained for research participants through the presentation of aggregate data in all presentations and publications.

4.3 Dissemination policy

Knowledge translation (KT) activities will be facilitated by the leadership of team investigators locally, nationally and internationally. CCKO represents a fully integrated KTE paradigm. The PI and co-PI have worked with patients and PCMCH to craft the provincial strategy, providing population-based ICES data to understand the target population (numbers/location of children, patterns of care, health system costs), and clinical expertise in complex care. The committees overseeing implementation and evaluation encompass key knowledge users (patients, families, clinicians, administrators), allowing for seamless KT. PCMCH is providing some evaluation support for this project and will be accountable to MOHLTC for the evaluation. Executive Summaries and/or Presentations will be shared for wide dissemination to a variety of organizations/collaboratives including provincial associations (e.g. OACRS, OACCAC, OHA) and the Canadian Association of Pediatric Health Centres Complex Care Community of Practice. Academic KT will occur through presentation at major health policy forums (e.g. CAHSPR) and publications in high-impact, peer-reviewed journals.

At the end of the study, a study summary will be shared on PCMCH's online portal, which describes the key findings of the intervention. A basic demographic overview would be provided (to give a sense of the study sample), as well as key findings related to service delivery outcomes and families' overall experience with the key workers and care plans.

References

1. Wilson-Costello D, Friedman H, Minich N, Fanaroff AA, Hack M. Improved survival rates with increased neurodevelopmental disability for extremely low birth weight infants in the 1990s. Pediatrics. 2005;115(4):997-1003.
2. Tennant PW, Pearce MS, Bythell M, Rankin J. 20-year survival of children born with congenital anomalies: a population-based study. Lancet. 2010;375(9715):649-56.
3. Plioplys AV. Survival rates of children with severe neurologic disabilities: a review. Seminars in pediatric neurology. 2003;10(2):120-9.
4. Wise PH. The transformation of child health in the United States: social disparities in child health persist despite dramatic improvements in child health overall. Health affairs. 2004;23(5):9-25.
5. Cohen E, Kuo DZ, Agrawal R, Berry JG, Bhagat SK, Simon TD, et al. Children with medical complexity: an emerging population for clinical and research initiatives. Pediatrics.127(3):529-38.
6. Cohen E, Berry JG, Camacho X, Anderson G, Wodchis W, Guttmann A. Patterns and costs of health care use of children with medical complexity. Pediatrics. 2012;130(6):e1463-70.
7. Neff JM, Sharp VL, Muldoon J, Graham J, Myers K. Profile of medical charges for children by health status group and severity level in a Washington State Health Plan. Health services research. 2004;39(1):73-89.
8. Simon TD, Berry J, Feudtner C, Stone BL, Sheng X, Bratton SL, et al. Children with complex chronic conditions in inpatient hospital settings in the United States. Pediatrics. 2010;126(4):647-55.
9. Dosa N, Boeing N, Ms N, Kanter R. Excess risk of severe acute illness in children with chronic health conditions. . Pediatrics. 2001;107(3):499-504.
10. Sacchetti A, Sacchetti C, Carraccio C, Gerardi. M. The potential for errors in children with special health care needs. Acad Emerg Med. 2000;7(11):1330-3.
11. Slonim A, LaFleur B, Ahmed W, Joseph. J. Hospital-reported medical errors in children. Pediatrics. 2003;111(3):617-21.
12. Matlow A, Wright J, Zimmerman B, Thomson K, Valente M. How can the principles of complexity science be applied to improve the coordination of care for complex pediatric patients? Qual Saf Health Care. 2006;15(2):85-8.
13. Raina P, O'Donnell M, Rosenbaum P, Brehaut J, Walter SD, Russell D, et al. The health and well-being of caregivers of children with cerebral palsy. Pediatrics. 2005;115(6):e626-36.
14. Brehaut JC, Kohen DE, Raina P, Walter SD, Russell DJ, Swinton M, et al. The health of primary caregivers of children with cerebral palsy: how does it compare with that of other Canadian caregivers? Pediatrics. 2004;114(2):e182-91.
15. Thorne SE, Radford MJ, Armstrong EA. Long-term gastrostomy in children: caregiver coping. Gastroenterol Nurs. 1997;20(2):46-53.
16. Cohen E, Friedman JN, Mahant S, Adams S, Jovcevska V, Rosenbaum P. The impact of a complex care clinic in a children's hospital. Child Care Health Dev. 2010;36(4):574-82.
17. Cohen E, Lacombe-Duncan A, Spalding K, MacInnis J, Nicholas D, Narayanan UG, et al. Integrated complex care coordination for children with medical complexity: a mixed-methods evaluation of tertiary care-community collaboration. BMC health services research. 2012;12:366.

18. Cohen E, Bruce-Barrett C, Kingsnorth S, Keilty K, Cooper A, Daub S. Integrated complex care model: lessons learned from inter-organizational partnership. Healthcare quarterly. 2011;14 Spec No 3:64-70.
19. Berry JG, Hall M, Neff J, Goodman D, Cohen E, Agrawal R, et al. Children with medical complexity and medicaid: spending and cost savings. Health affairs. 2014;33(12):2199-206.
20. Cohen E, Jovcevska V, Kuo DZ, Mahant S. Hospital-based comprehensive care programs for children with special health care needs: a systematic review. Archives of pediatrics & adolescent medicine. 2011;165(6):554-61.
21. Ontario Medical Association. Health Links. [cited 2016 Mar 20] Availabe from: https://www.oma.org/benefits/healthlinks/pages/default.aspx
22. Brown CA, Lilford RJ. The stepped wedge trial design: a systematic review. BMC Medical Research Methodology. 2006;6:54.
23. Hussey MA, Hughes JP. Design and analysis of stepped wedge cluster randomized trials. Contemporary Clinical Trials. 2007;28:192-191.
24. Chapter 2. What is Care Coordination? June 2014. Agency for Healthcare Research and Quality, Rockville, MD. http://www.ahrq.gov/professionals/prevention-chronic-care/improve/coordination/atlas2014/chapter2.html
25. Mangione-Smith. Family Experiences with Care Coordination measure set (FECC) 2015 [cited 2016]. Available from: http://www.ahrq.gov/sites/default/files/wysiwyg/policymakers/chipra/factsheets/chipra_15-p002-ef.pdf.
26. Rita Mangione-Smith, personal communication: 2016 Jan 12.
27. World Health Organization. Consitution of WHO: principles [cited Mar 2016]. Available from: http://www.who.int/about/mission/en/
28. Ravens-Sieberer U, Gosch A, Rajmil L, Erhart M, Bruil J, Duer W, et al. KIDSCREEN-52 quality-of-life measure for children and adolescents. Expert Review of Pharmacoeconomics & Outcomes Research. 2005;5(3):353-64.
29. Fayed N, De Camargo OK, Kerr E, Rosenbaum P, Dubey A, Bostan C, et al. Generic patient-reported outcomes in child health research: a review of conceptual content using World Health Organization definitions. Developmental Medicine & Child Neurology. 2012;54(12):1085-95.
30. Brand K, Court C. Pain assessment in children. Anaesthesia & Intensive Care Medicine. 2010;11(6):214-6.
31. McGrath PA. An assessment of children's pain: a review of behavioral, physiological and direct scaling techniques. Pain. 1987;31(2):147-76.
32. Diener E. Subjective well-being: The science of happiness and a proposal for a national index. American psychologist. 2000;55(1):34.
33. Diener E, Emmons RA, Larsen RJ, Griffin S. The satisfaction with life scale. Journal of personality assessment. 1985;49(1):71-5.
34. Cella D, Yount S, Rothrock N, Gershon R, Cook K, Reeve B, et al. The Patient-Reported Outcomes Measurement Information System (PROMIS): progress of an NIH Roadmap cooperative group during its first two years. Medical care. 2007;45(5 Suppl 1):S3.
35. Lai J-S, Stucky BD, Thissen D, Varni JW, DeWitt EM, Irwin DE, et al. Development and psychometric properties of the PROMIS® pediatric fatigue item banks. Quality of Life Research. 2013;22(9):2417-27.
36. Sprangers MA. Response-shift bias: a challenge to the assessment of patients' quality of life in cancer clinical trials. Cancer Treat Rev. 1996; 22 Suppl A: 55–62.

37. Rapkin BD, Schwartz CE. Toward a theoretical model of quality of life appraisal: Implication of findings from studies of response shift. Health and Quality of Life Outcomes. 2004; 2:14.
38. Nolte S, Elsworth GR, Sinclair AJ, Osborne RH. The inclusion of 'then-test' questions in post-test questionnaires alters post-test responses: a randomized study of bias in health program evaluation. Qual Life Res. 2011; 21(3):487-94.
39. Jaeschke R, Singer J, Guyatt GH. Measurement of health status: ascertaining the minimal clinically important difference. Controlled clinical trials. 1989;10(4):407-15.

Appendix 1 – Strategic Framework for CCKO

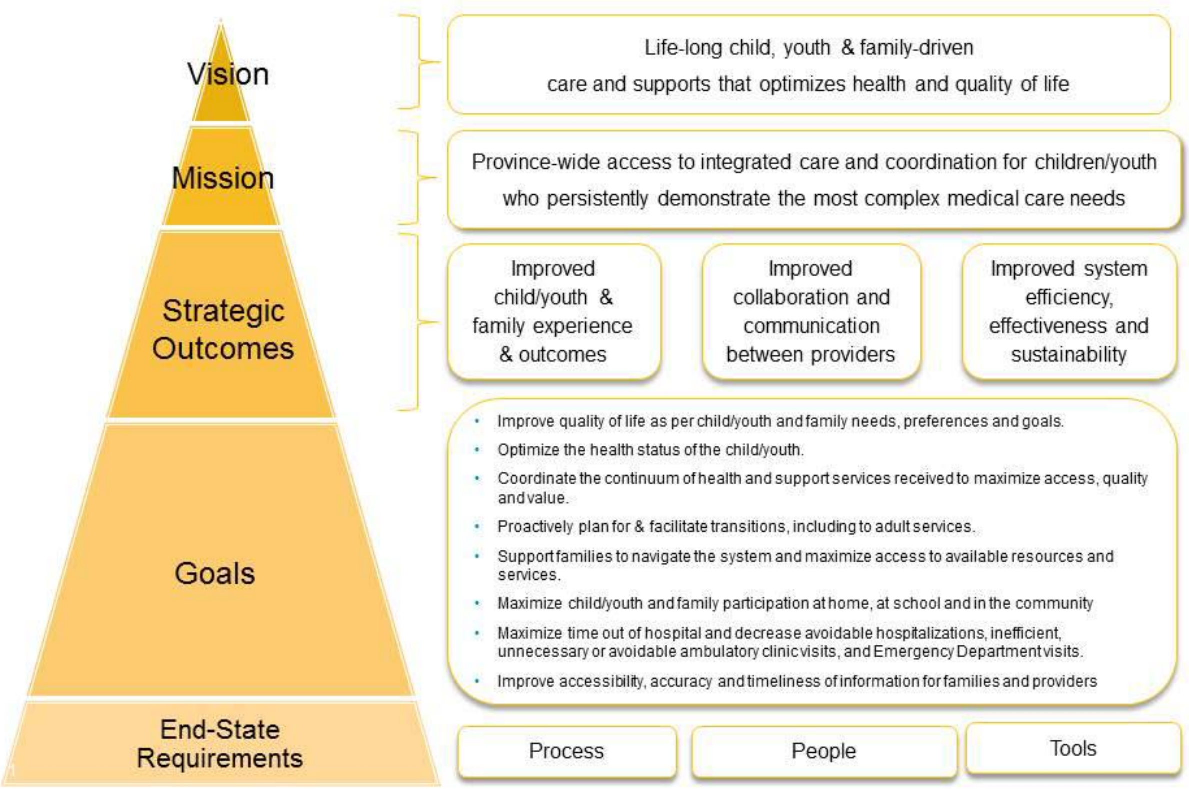

Appendix 2 – CCKO Planning and Implementation Regions

Region	LHINs Included	Primary Tertiary Hospital Serving Target Population	Children with persistent high health service utilization	
			Estimated # in Region	% of total population
1	Erie St. Clair & South West	London Health Sciences	885	14.1%
2	Hamilton Niagara Haldimand Brant & Waterloo Wellington	Hamilton Health Sciences	1118	17.8%
3	Toronto Central, Mississauga Halton, Central West, Central, Central East & North Simcoe Muskoka	Hospital for Sick Children	3064	48.6%
4	Champlain & South East	Children's Hospital of Eastern Ontario	823	13.1%
5	North East & North West	Mixed	400	6.4%
