# Supplementary material for: Effectiveness of Structured Care Coordination for Children With Medical Complexity: The Complex Care for Kids Ontario (CCKO) Randomized Clinical Trial
Source: JAMA Pediatr. 2023 Mar 20;177(5):461–71. doi: 10.1001/jamapediatrics.2023.0115 (PMC10028546; doi:10.1001/jamapediatrics.2023.0115)
Supplement: Supplement 2. — eFigure 1. Primary and Secondary Outcome Schematic eFigure 2. Comparison of study primary outcomes between intervention and waitlist groups after multiple imputation eFigure 3. Comparison of parent-reported study secondary outcomes between intervention and waitlist groups after multiple imputation eTable 1. CCKO regional and community complex care clinics and breakdown of participants from each site eTable 2. Baseline characteristics of participants with complete health administrative data eTable 3. Baseline characteristics of participants with and without loss to follow-up at 12 and 24 months eTable 4. Caregiver-reported secondary outcomes between the intervention and waitlist group at baseline, month 12 and month 24 follow-up eTable 5. Individual Family Experiences with Care Coordination (FECC) outcomes at baseline, month 12 and month 24 follow-up eTable 6. Caregiver-reported secondary outcomes between the intervention and waitlist group at baseline, month 12 and month 24 follow-up with imputed missing data (multiple imputation) eTable 7. Individual Family Experiences with Care Coordination (FECC) outcomes at baseline, month 12 and month 24 follow-up with imputed missing data (multiple imputation) eTable 8. Health service cost and utilization among study participants using the initial complex care clinic visit date as the start of the intervention eTable 9. Health service cost and utilization among study participants who had their entire Year 1 and Year 2 follow-up before estimated onset date of the pandemic (March 13, 2020) eTable 10. Health service cost and utilization among study participants, excluding deaths in Year 1 and Year 2 eTable 11. Health service cost and utilization among study participants who had their entire 1st and 2nd year follow-up before estimated onset date of the pandemic (March 13, 2020), excluding deaths in Year 1 and Year 2 [file jamapediatr-e230115-s002.pdf]

Supplemental Online Content

Cohen E, Quartarone S, Orkin J, et al. Effectiveness of structured care coordination for children with medical complexity: the Complex Care for Kids Ontario (CCKO) randomized clinical trial. *JAMA Pediatr*. Published online March 20, 2023. doi:10.1001/jamapediatrics.2023.0115

eFigure 1. Primary and Secondary Outcome Schematic

eFigure 2. Comparison of study primary outcomes between intervention and waitlist groups after multiple imputation

eFigure 3. Comparison of parent-reported study secondary outcomes between intervention and waitlist groups after multiple imputation

eTable 1. CCKO regional and community complex care clinics and breakdown of participants from each site

eTable 2. Baseline characteristics of participants with complete health administrative data

eTable 3. Baseline characteristics of participants with and without loss to follow-up at 12 and 24 months

eTable 4. Caregiver-reported secondary outcomes between the intervention and waitlist group at baseline, month 12 and month 24 follow-up

eTable 5. Individual Family Experiences with Care Coordination (FECC) outcomes at baseline, month 12 and month 24 follow-up

eTable 6. Caregiver-reported secondary outcomes between the intervention and waitlist group at baseline, month 12 and month 24 follow-up with imputed missing data (multiple imputation)

eTable 7. Individual Family Experiences with Care Coordination (FECC) outcomes at baseline, month 12 and month 24 follow-up with imputed missing data (multiple imputation)

eTable 8. Health service cost and utilization among study participants using the initial complex care clinic visit date as the start of the intervention

eTable 9. Health service cost and utilization among study participants who had their entire Year 1 and Year 2 follow-up before estimated onset date of the pandemic (March 13, 2020)

eTable 10. Health service cost and utilization among study participants, excluding deaths in Year 1 and Year 2

eTable 11. Health service cost and utilization among study participants who had their entire 1st and 2nd year follow-up before estimated onset date of the pandemic (March 13, 2020), excluding deaths in Year 1 and Year 2

This supplemental material has been provided by the authors to give readers additional information about their work.

© 2023 Cohen E et al. *JAMA Pediatrics.*

eFigure 1: Primary and secondary outcomes schematic

3 Co-Primary Outcomes

Service Delivery Outcomes (Measurement Tool)	Child Outcomes (Measurement Tool)	Parent Outcomes (Measurement Tool)	Health System Outcomes (Measurement Tool)
Coordination of Care Among Health Care Professionals (FECC) [38]	Quality of Life (KIDSCREEN) [39,41]	Physical Health (PROMIS) [44,45]	Healthcare service use
Coordination of Care Between Health Care Professionals and Families (FECC) [38]	Emotional Health (KIDSCREEN) [39,41]	Mental Health (PROMIS) [44,45]	Healthcare service costs
Utility of Care Planning Tools (FECC) [38]	Physical Pain (VAS) [39,41]	Fatigue (PROMIS) [44,45]	
Individual family experiences with care coordination (FECC) [38]		Sleep Disturbance (PROMIS) [44,45]	
		Satisfaction with Life Scale (SWLS) [41,42]	

Acronyms:
FECC, Family Experiences with Coordination of Care
PROMIS, Patient Reported Outcomes Measurement Information System
SWLS, Satisfaction with Life Scale
VAS, Visual Analog Scale

© 2023 Cohen E et al. *JAMA Pediatrics.*

eFigure 2: Comparison of study primary outcomes between intervention and waitlist groups after multiple imputation.

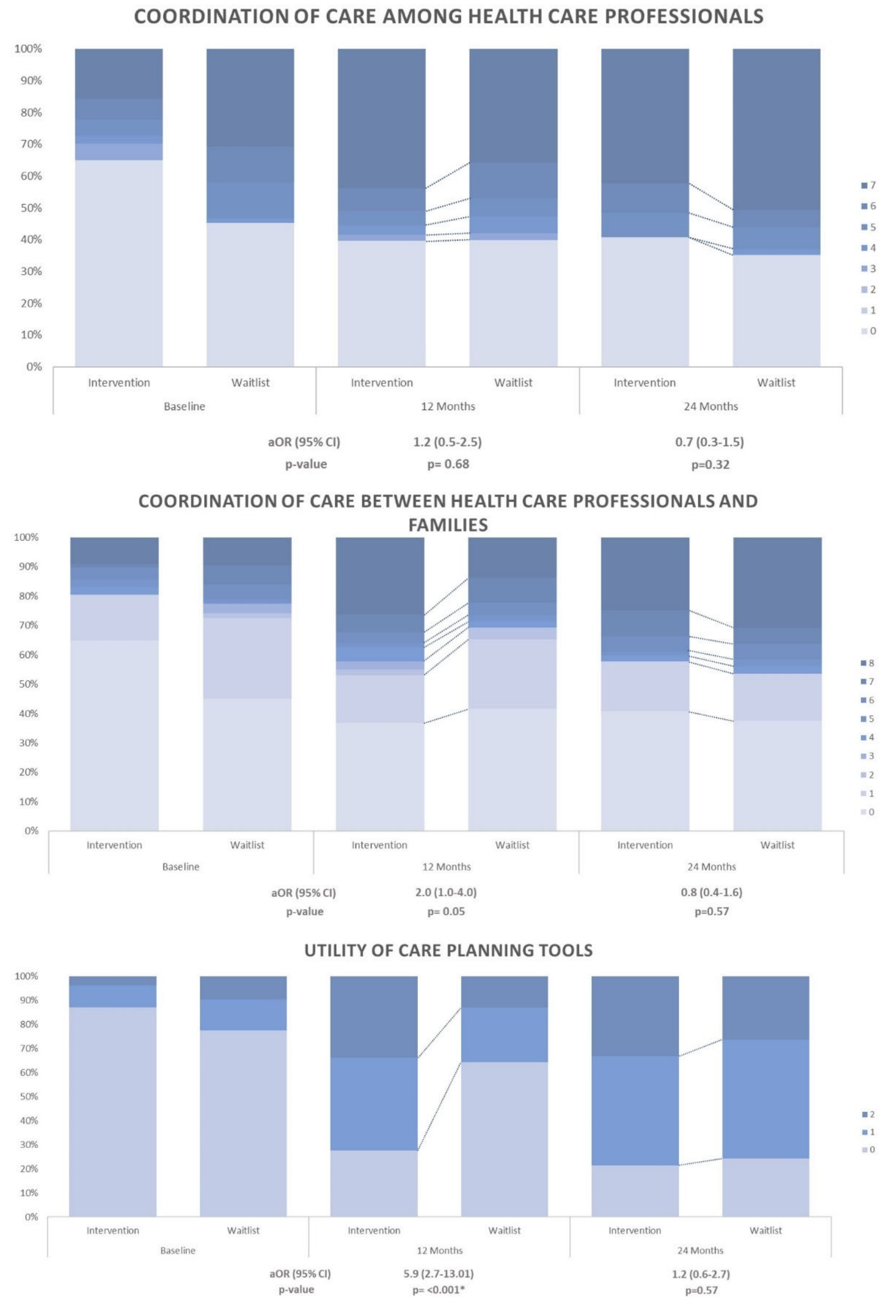

Family Experience with Care Coordination (FECC) scores range 0-7, 0-8 and 0-2, based on the sum scores of FECC 8a-8b (Coordination of Care Among Health Providers), FECC 5 (Coordination of Care Between Health Providers and Families and FECC 16-17 (Utility of Care Planning Tools) indicators respectively, where larger values indicate better care. All p-values are calculated based on comparisons of outcomes at the end of 12 months (Year 1) and 24 months (Year 2) using ordinal regression for FECC indicators. All models included center as a random intercept and are adjusted for baseline values and expressed as an adjusted odds ratio (aOR).

© 2023 Cohen E et al. *JAMA Pediatrics.*

eFigure 3: Comparison of parent-reported study secondary outcomes between intervention and waitlist groups after multiple imputation.

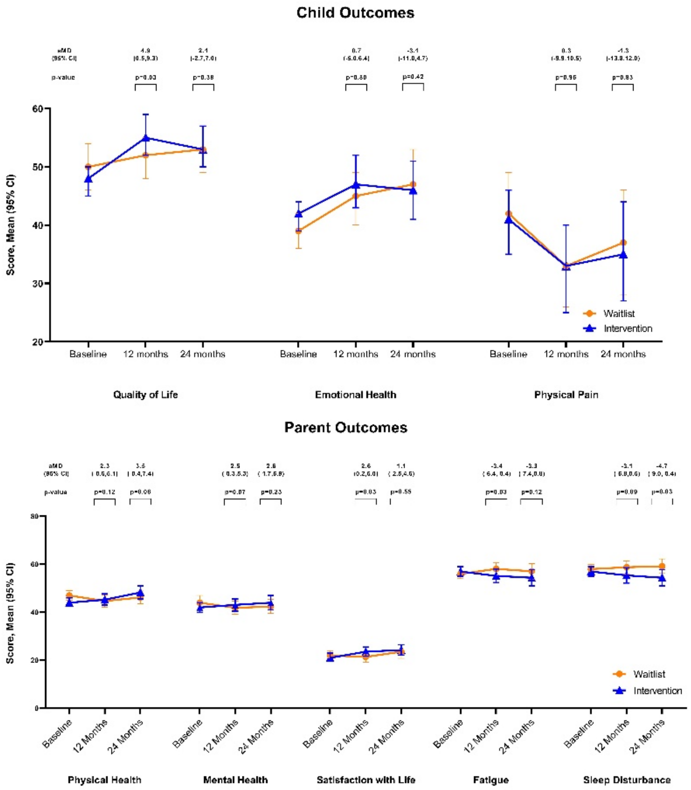

Outcomes include child outcomes and parental caregiver outcomes. Child outcomes include quality of life, emotional health, measured by parent-proxy respectively using subscales from the KIDSCREEN-52,, and physical pain measured with a 10 cm linear Visual Analog Scale [VAS][43,44]). Caregiver outcomes include: perceived physical health, mental health, fatigue and sleep disturbance (measured by the Patient Reported Outcomes Measurement Information System (PROMIS)), and satisfaction with life, measured with the Satisfaction with Life Scale [SWLS].[47,48] Higher scores indicate more of the outcome of interest which can be improvement (parental physical health, mental health and satisfaction with life; child quality of life and emotional health) or deterioration (parent fatigue and sleep disturbance, child physical pain) depending on the outcome. All outcomes are modelled using linear regression including center as a random intercept and are adjusted for baseline values and expressed as an adjusted mean difference (aMD). Asterisks represent statistical significance using thresholds based on the Holm-Bonferroni method to adjust for false positives, where the overall target type I error rate was 0.05.

© 2023 Cohen E et al. *JAMA Pediatrics.*

eTable 1: CCKO regional and community complex care clinics and breakdown of participants from each site

		Randomized (N=207)		Included (N=144)	
**Site Name**	**Location**	**Intervention** (N=105)	**Waitlist** (N=102)	**Intervention** (N=81)	**Waitlist** (N=63)
The Hospital for Sick Children (Regional CCKO Lead)	Toronto, ON	32	35	25	24
Michael Garron Hospital	Toronto, ON	3	3	1	2
North York General Hospital	Toronto, ON	18	15	15	7
Credit Valley Hospital	Mississauga, ON	8	9	4	6
Peterborough Regional Health Centre	Peterborough, ON	6	6	5	4
Royal Victoria Hospital	Barrie, ON	4	4	4	3
Orillia Soldiers' Memorial Hospital	Orillia, ON	6	7	5	6
Children's Hospital of Eastern Ontario (Regional CCKO Lead) including Cochrane Temiskaming Children's Treatment Centre and Monfort Hospital	Ottawa, ON	17	14	14	6
McMaster Children's Hospital (Regional CCKO Lead) including Niagara Children's Centre	Hamilton, ON	11	9	8	5

© 2023 Cohen E et al. *JAMA Pediatrics.*

eTable 2: Baseline characteristics of participants with complete health administrative data.

	Year 1 [a]		Year 2 [b]	
**Characteristic**	**Intervention** (N=79)	**Waitlist** (N=62)	**Intervention** (N=76)	**Waitlist** (N=59)
**Age, mean (SD), y**	3.9 (4.8)	4.3 (4.3)	3.9 (4.8)	4.2 (4.2)
**Sex**				
Male	48 (61)	38 (61)	47 (62)	37 (63)
**Deprivation Quintile [c]**				
1	16 (20)	6 (10)	14 (18)	6 (10)
2	19 (24)	18 (29)	19 (25)	18 (31)
3	11 (14)	11 (18)	10 (13)	11 (19)
4	15 (19)	11 (18)	15 (20)	10 (17)
5	16 (20)	16 (26)	16 (21)	14 (24)
**Rurality**				
Yes	9 (11)	7 (11)	9 (12)	7 (12)
**Complex Chronic Conditions**	74 (94)	56 (90)	71 (93)	53 (90)
**Complex Chronic Condition Organ Involvement**				
Multi	51 (65)	39 (63)	49 (65)	38 (64)
Single	23 (29)	17 (27)	22 (29)	15 (25)
**Diagnosis**				
Neuromuscular	45 (57)	32 (52)	43 (57)	29 (49)
Cardiovascular	32 (41)	19 (31)	31 (41)	19 (32)
Respiratory	7 (9)	9 (15)	<=5	9 (15)
Renal	12 (15)	<=5	11 (15)	<=5
Gastrointestinal	40 (51)	31 (50)	38 (50)	31 (53)
Hematologic/ Immunologic	<=5	<=5	<=5	<=5
Metabolic	12 (15)	7 (11)	12 (16)	6 (10)
Congenital	19 (24)	13 (21)	18 (24)	13 (22)
Neoplasms	<=5	<=5	<=5	<=5
Miscellaneous	10 (13)	11 (18)	9 (12)	11 (19)
**Health service cost (CAD)**			-	-
Total cost, median (IQR)	49,970 (20,530-108,741)	47,834 (22,310-131,930)	-	-

All data are presented as a number (%) unless otherwise indicated.

[a] total N=141 among the 144 eligible as 3 participants did not have a valid identifier to link the administrative data.

[b] total N=135 for Year 2 analysis as 6 participants (3 in intervention group and 3 in waitlist group) died by the end of Year 1.

[c] Deprivation quintile refers to a geographically based index developed to quantify the degree of marginalization occurring across the province of Ontario. It is comprised of 4 major dimensions of marginalization: residential instability, material deprivation, dependency, and ethnic diversity. Missing data suppressed due to small (<=5) cell size.

© 2023 Cohen E et al. *JAMA Pediatrics.*

eTable 3: Comparison of baseline characteristics and health administrative data of participants with LTFU at 12 and 24 months.

	LTFU or withdrew by 12 months		Had response at 12 months (Not LFTU)		LTFU or withdrew by 24 months		Had response at 24 months (Not LFTU)	
	Intervention N=10	Waitlist N=6	Intervention N=64	Waitlist N=53	Intervention N=13	Waitlist N=10	Intervention N=60	Waitlist N=48
**Demographic Characteristics**								
Of the Child								
**Age in months, mean (SD)**	42 (39)	77 (58)	57 (62)	50 (50)	43.6 (50.0)	80.3 (53.2)	58.2 (62.6)	48.0 (49.9)
Birth-12 mo.	2 (20)	1 (17)	22 (34)	15 (28)	3 (23.1)	2 (20)	20 (33.3)	14 (29.2)
13 mo-2 y	2 (20)	0	7 (11)	9 (17)	2 (15.4)	0	7 (11.7)	9 (18.8)
2-5 y	4 (40)	2 (33)	14 (22)	11 (21)	5 (38.5)	2 (20.0)	13 (21.7)	10 (20.8)
6-11 y	2 (20)	2 (33)	10 (16)	14 (26)	3 (23.1)	5 (50.0)	9 (15)	11 (22.9)
12-18 y	0	1 (17)	11 (17)	4 (8)	00	1 (10.0)	11 (18.3)	4 (8.3)
**Sex**								
Male	5 (50)	2 (33)	39 (61)	35 (66)	8 (61.5)	5 (50.0)	35 (58.3)	32 (66.7)
**Self-reported ethnicity**								
African	1 (10)	1 (17)	2 (3)	1 (2)	2 (15.4)	1 (10.0)	0	1 (2.1)
Asian	0	1 (17)	5 (8)	1 (2)	0	1 (10.0)	5 (8.3)	1 (2.1)
Canadian	5 (50)	1 (17)	21 (33)	22 (42)	6 (46.2)	2 (20.0)	20 (33.3)	20 (41.7)
Caribbean/West Indian	1 (10)	0	1 (2)	0	1 (7.7)	0	1 (1.67)	0
European	1 (10)	0	3 (5)	4 (8)	1 (7.7)	0	3 (5.0)	4 (8.3)
South Asian	0	1 (17)	5 (8)	5 (9)	1 (7.7)	2 (20.0)	4 (6.7)	4 (8.3)
Don't Know	1 (10)	2 (33)	27 (42)	20 (38)	1 (7.7)	4 (40.0)	27 (45.0)	18 (37.5)
Unspecified	1 (10)	0	0	0	1 (7.7)	0	0	0
**Enrolled in school outside the home**								
Yes	2 (20)	3 (50)	23 (36)	19 (36)	3 (23.1)	6 (60.0)	22 (36.7)	16 (33.3)
**School days missed per month, mean (SD)**	10 (9), N=3	9 (7), N=3	5 (3), N=23	7 (5), N=17	8.7 (7.5), N=4	9.2 (4.2), N=6	5.0 (3.0), N=22	6.9 (5.6), N=14
**Clinical baseline characteristics**								
**Primary diagnoses**	N=10	N=5	N=64	N=53	N= 13	N= 9	N= 60	N= 48
Neurologic/ neuromuscular	5 (50)	5 (100)	28 (44)	25 (47)	7 (53.9)	8 (88.9)	26 (43.3)	22 (45.8)
Cardiovascular	0	0	3 (5)	4 (8)	0	0	3 (5.0)	4 (8.3)
Respiratory	0	0	0	2 (4)	0	1 (11.1)	0	1 (2.1)

© 2023 Cohen E et al. *JAMA Pediatrics.*

	LTFU or withdrew by 12 months		Had response at 12 months (Not LFTU)		LTFU or withdrew by 24 months		Had response at 24 months (Not LFTU)	
	**Intervention N=10**	**Waitlist N=6**	**Intervention N=64**	**Waitlist N=53**	**Intervention N=13**	**Waitlist N=10**	**Intervention N=60**	**Waitlist N=48**
Renal/ urologic	2 (20)	0	1 (2)	1 (2)	2 (15.4)	0	1 (1.7)	1 (2.1)
Gastrointestinal	1 (10)	0	0	1 (2)	1 (7.7)	0	0	1 (2.1)
Hematologic/ immunologic	0	0	0	1 (2)	0	0	0	1 (2.1)
Metabolic	0	0	0	2 (4)	0	0	3 (5.0)	2 (4.2)
Congenital/ genetic defect	2 (20)	0	21 (33)	14 (26)	3 (23.1)	0	19 (31.7)	13 (27.1)
Premature/ neonatal	0	0	3 (5)	3 (6)	0	0	3 (5.0)	3 (6.4)
Malignancy	0	0	2 (3)	0	0	0	2 (3.3)	0
Miscellaneous	0	0	3 (5)	0	0	0	3 (5.0)	0
**No. of diagnoses, mean (SD)**	8 (3)	5 (3)	7 (4)	6 (3)	8.2 (4.0)	6.1 (3.5)	6.7 (3.4)	6.0 (3.0)
**Medications used, mean (SD)**	7 (5)	3 (3)	5 (4)	5 (3)	6.8 (4.8)	5.0 (4.6)	5.3 (3.8)	4.4 (3.1)
**Technology devices used, mean (SD)**	3 (2)	2 (3)	3 (2)	2 (2)	2.2 (1.9)	2.4 (2.0)	2.8 (2.0)	2.3 (1.8)
**Technology devices used**								
Feeding devices [d]	7 (70)	3 (50)	46 (72)	43 (81)	9 (69.2)	6 (60.0)	43 (71.7)	39 (81.3)
Respiratory devices [e]	5 (50)	1 (17)	20 (31)	14 (26)	6 (46.2)	4 (40.0)	18 (30.0)	11 (22.9)
Mobility devices [f]	1 (10)	2 (33)	29 (45)	22 (42)	2 (12.4)	4 (40.0)	28 (46.7)	19 (39.6)
Other	4 (40)	2 (33)	30 (47)	11 (21)	4 (30.8)	3 (30.0)	30 (50.0)	10 (20.8)
**Diet**								
Oral	3 (30)	3 (50)	22 (34)	11 (21)	4 (30.8)	4 (40.0)	21 (35.0)	10 (20.8)
Enterostomy tube	7 (70)	3 (50)	36 (56)	34 (64)	8 (61.5)	5 (50.0)	34 (56.7)	31 (64.6)
Oral and enterostomy tube	0	0	6 (9)	8 (15)	1 (7.7)	1 (10.0)	5 (8.3)	7 (14.6)
**Communication skills-age ≥ 12 months**	N=8	N=3	N=39	N=34	N=10	N=6	N=37	N=30
Verbal	3 (38)	0	8 (21)	4 (12)	3 (30.0)	0	8 (21.6)	4 (13.3)
Non-verbal	5 (63)	3 (100)	31 (79)	30 (88)	7 (70.0)	6 (100)	29 (78.4)	26 (86.7)
**Gross Motor Functioning, age ≥ 24 months**								
**Difficulties with Gross Motor Functioning j, [k]**	N=4	N=2	N=21	N=21	N=5	N=4	N=20	N=18

© 2023 Cohen E et al. *JAMA Pediatrics.*

	LTFU or withdrew by 12 months		Had response at 12 months (Not LFTU)		LTFU or withdrew by 24 months		Had response at 24 months (Not LFTU)	
	Intervention N=10	Waitlist N=6	Intervention N=64	Waitlist N=53	Interventio n N=13	Waitlist N= 10	Intervention N=60	Waitlist N=48
Level 1	0	0	2 (10)	0	0	0	2 (10.0)	0
Level 2	0	0	2 (10)	3 (14)	0	0	2 (10.0)	3 (16.7)
Level 3	1 (25)	0	1 (5)	1 (5)	1 (20.0)	0	1 (5.0)	1 (5.6)
Level 4	0	1 (50)	5 (24)	0	0	1 (25.0)	5 (25.0)	0
Level 5	2 (50)	1 (50)	6 (29)	10 (48)	3 (60.0)	2 (50.0)	5 (25.0)	9 (50.0)
Level 6	1 (25)	0	5 (24)	7 (33)	1 (20.0)	1 (25.0)	5 (25.0)	5 (27.8)
Of the Parental Caregiver Respondent								
**Age in years, mean (SD)**	38 (8), N=2	36 (10), N=6	34 (7), N=42	36 (9), N=35	35 (8.9), N=4	38.4 (11.1), N=8	33.6 (7.1), N=40	35.6 (8.4), N=32
**Sex**								
Male	6 (60)	5 (83)	54 (84)	48 (91)	7 (53.9)	9 (90.0)	52 (86.6)	43 (89.6)
**Marital status**								
Married/ common law	8 (80)	3 (50)	55 (86)	41 (77)	10 (76.9)	5 (50.0)	52 (86.7)	38 (79.2)
Single	2 (20)	3 (50)	6 (9)	5 (9)	2 (15.4)	4 (40.0)	6 (10.0)	4 (8.3)
Widowed	0	0	0	1 (2)	0	1 (10.0)	0	0
Separated/ divorced	0	0	3 (5)	6 (11)	1 (7.7)	0	2 (3.3)	6 (12.5)
**Employment status**								
Employed full-time	3 (30)	1 (17)	25 (39)	18 (34)	4 (30.8)	2 (20.0)	24 (40.0)	17 (35.4)
Employed part-time	2 (20)	1 (17)	12 (19)	3 (6)	2 (15.4)	1 (10.0)	12 (20.0)	3 (6.3)
Unemployed/ homemaker	3 (30)	1 (17)	22 (34)	23 (43)	5 (38.5)	2 (20.0)	19 (31.7)	21 (43.8)
Receiving social assistance/ disability/ pension	2 (20)	2 (33)	4 (6)	6 (11)	2 (15.4)	3 (30.0)	4 (6.7)	5 (10.4)
Student	0	1 (17)	1 (2)	3 (6)	0	2 (20.0)	1 (1.7)	2 (4.2)
**Education level**								
Elementary school (some or completed)	0	0	1 (2)	0	0	0	1 (1.7)	0
Some secondary/ high school	2 (20)	0	1 (2)	2 (4)	2 (15.4)	0	1 (1.7)	2 (4.2)
Completed secondary/ high school	3 (30)	0	3 (5)	6 (11)	3 (23.1)	2 (20.0)	3 (5.0)	4 (8.3)
Some post-secondary	2 (20)	3 (50)	15 (23)	7 (13)	2 (15.4)	5 (50.0)	15 (25.0)	5 (10.4)
Received university or college degree	3 (30)	3 (50)	44 (69)	38 (72)	6 (46.2)	3 (30.0)	40 (66.7)	37 (77.1)

© 2023 Cohen E et al. *JAMA Pediatrics.*

eTable 4: Caregiver-reported secondary outcomes between the intervention and waitlist group at baseline, month 12 and month 24 follow-up

	Intervention			Waitlist			Month 12 [a]		Month 24 [b]	
	**Baseline**	**Month 12**	**Month 24**	**Baseline**	**Month 12**	**Month 24**	**aMD (95% CI)**	**P Value [d]**	**aMD (95% CI)**	**P Value [d]**
**Secondary Outcomes- Child measures**										
Quality of Life [e]	47.8 (45.4 to 50.2)	54.5 (51.4 to 57.6)	53.2 (50.2 to 56.2)	50.1 (50.4 to 56.2)	51.1 (47.8 to 54.4)	51.8 (48.4 to 55.2)	4.0 (0.4 to 7.6)	0.03	2.0 (-1.6 to 5.6)	0.28
Emotional Health [f]	41.7 (39.4 to 44.0)	46.2 (42.7 to 49.7)	47.1 (43.7 to 50.5)	39.1 (36.3 to 42.0)	44.6 (41.0 to 48.2)	47.8 (43.6 to 52.0)	-0.7 (-5.1 to 3.6)	0.74	-3.2 (-7.8 to 1.3)	0.16
Physical Pain [g]	40.8 (35.4 to 46.2)	32.1 (25.7 to 38.5)	35.2 (28.3 to 42.1)	42.3 (49.4 to 35.2)	31.9 (25.1 to 38.7)	35.2 (28.2 to 42.1)	0.8 (-7.7 to 9.3)	0.86	0.8 (-8.4 to 10.0)	0.87
**Secondary Outcomes- Parent measures**										
Physical Health [h]	44.4 (42.7 to 46.1)	45.1 (42.8 to 47.4)	47.1 (44.8 to 49.4)	46.8 (44.6 to 49.0)	44.9 (42.5 to 47.4)	45.6 (43.1 to 48.1)	1.8 (-0.7 to 4.4)	0.16	3.4 (0.7 to 6.0)	0.01*
Mental Health [i]	42.0 (39.9 to 44.1)	43.1 (40.6 to 45.6)	42.9 (40.4 to 45.4)	44.0 (41.2 to 46.8)	41.9 (39.2 to 44.6)	42.3 (39.8 to 44.9)	2.7 (0.3 to 5.1)	0.03	2.1 (-0.7 to 5.0)	0.14
Satisfaction with Life [l]	21.4 (19.9 to 22.9)	23.5 (21.9 to 25.1)	24.0 (21.9 to 26.1_	22.2 (20.2 to 24.2)	21.9 (19.7 to 24.1)	23.4 (21.5 to 25.3)	2.3 (0.1 to 4.5)	0.04	1.3 (-1.1 to 3.7)	0.29
Fatigue [j]	57.1 (55.2 to 59.0)	56.0 (53.3 to 58.7)	54.6 (51.6 to 57.6)	56.4 (54.0 to 58.9)	58.1 (55.5 to 60.7)	57.4 (54.5 to 60.3)	-2.6 (-5.2 to 0.0)	0.05	-4.1 (-7.2 to -1.0)	0.009*
Sleep Disturbance [k]	57.1 (55.2 to 59.0)	56.1 (53.3 to 58.9)	54.9 (52.0 to 57.8)	57.5 (55.3 to 59.7)	59.1 (56.4 to 61.8)	59.7 (57.2 to 62.2)	-2.8 (-6.0 to 0.3)	0.08	-5.2 (-8.6 to -1.7)	0.003*

Abbreviations: aMD, adjusted mean difference; CI, confidence interval
All data presented as mean (95% CI).
[a] aMD (95% CI), p value restricted to participants with completed baseline and month 12 follow up data
[b] aMD (95% CI), p value restricted to participants with completed baseline and month 24 follow up data
[c] aMD (95% CI), calculated as intervention and waitlist using linear regression
[d] P values were adjusted for multiple comparisons using the Holm-Bonferroni test where the overall target type 1 error rate was 0.05. Asterisk represents meeting significance threshold. At 12 months the threshold for the secondary outcomes-child measures were 0.02 for quality of life, 0.03 for emotional health, and 0.05 for physical pain, for the parent measures were 0.05 for physical health, 0.01 for mental health, 0.02 for fatigue, 0.03 for sleep disturbance and 0.01 for satisfaction with life. At 24 months the threshold for the secondary outcomes-child measures were 0.03 for quality of life, 0.02 for emotional health, and 0.05 for physical pain, and for the parent measures were 0.02 for physical health, 0.03 for mental health, 0.01 for fatigue, 0.01 for sleep disturbance and 0.05 for satisfaction with life
[e] Baseline, Intervention (n=72) and Waitlist (n=60); Month 6, Intervention (n=66) and Waitlist (n=53); Month 12 Intervention (n=63) and Waitlist (n=52); Month 24 Intervention (n=60) and Waitlist (n=46), KIDSCREEN Feeling subscale t-scores are standard scores based on a general pediatric population ranging from 0-100, mean=50, and 1SD=10

© 2023 Cohen E et al. *JAMA Pediatrics.*

[f] Baseline, Intervention (n=75) and Waitlist (n=59); Month 6, Intervention (n=65) and Waitlist (n=52); Month 12, Intervention (n=62) and Waitlist (n=51); Month 24, Intervention (n=59) and Waitlist (n=46): KIDSCREEN General Mood subscale t-scores are standard scores based on a general pediatric population ranging from 0-100, mean=50, and 1SD=10
[g] Baseline, Intervention (n=76) and Waitlist (n=61); Month 6, Intervention (n=68) and Waitlist (n=53); Month 12, Intervention (n=63) and Waitlist (n=52); Month 24, Intervention (n=60) and Waitlist (n=48); Visual analogue pain scale 0 =no hurt to 10 = extreme pain score transformed from 0-100.
[h] Baseline, Intervention (n=77) and Waitlist (n=62); Month 6 Intervention (n=67) and Waitlist (n=55); Month 12, Intervention (n=62) and Waitlist (n=53); Month 24, Intervention (n=60) and Waitlist (n=47): PROMIS Global-10 Physical Health subscale t-scores are standard scores based on a general adult population ranging from 0-100, mean=50, and 1SD=10
[i] Baseline, Intervention (n=77) and Waitlist (n=62); Month 6, Intervention (n=67) and Waitlist (n=55); Month 12, Intervention (n=62) and Waitlist (n=53); Month 24, Intervention (n=60) and Waitlist (n=47); PROMIS Global-10 Emotional Health subscale t-scores are standard scores based on a general adult population ranging from 0-100, mean=50, and 1SD=10
[j] Baseline, Intervention (n=77) and Waitlist (n=62); Month 6, Intervention (n=69) and Waitlist (n=54); Month 12, Intervention (n=64) and Waitlist (n=53); Month 24, Intervention (n=60) and Waitlist (n=48); Diener's Satisfaction with Life Scale raw sum score range=5-35.
[k] Baseline, Intervention (n=77) and Waitlist (n=62); Month 6, Intervention (n=67) and Waitlist (n=55); Month 12, Intervention (n=62) and Waitlist (n=53); Month 24, Intervention (n=60) and Waitlist (n=48); PROMIS Fatigue t-scores are standard scores based on a general adult population ranging from 0-100, mean=50, and 1SD=10
[l] Baseline, Intervention (n=77) and Waitlist (n=62); Month 6, Intervention (n=67) and Waitlist (n=55); Month 12, Intervention (n=62) and Waitlist (n=53); Month 24, Intervention (n=60) and Waitlist (n=48); PROMIS Sleep Disturbance t-scores are standard scores based on a general adult population ranging from 0-100, mean=50, and 1SD=10

© 2023 Cohen E et al. *JAMA Pediatrics.*

eTable 5: Individual Family Experiences with Care Coordination (FECC) outcomes baseline, month 12 and month 24 follow-up

		Baseline		Month 12				Month 24			
**Indicator ID [a]**	**Indicator Question**	**Intervention (N=77)**	**Waitlist (N=62)**	**Intervention (N=64)**	**Waitlist (N=53)**	**aMD (95% CI)**	**P Value**	**Intervention (N=60)**	**Waitlist (N=48)**	**aMD (95% CI)**	**P Value**
**FECC-1**	Did the main provider/team help you to manage your child's care or treatment from different doctors and care providers?	41.3 (31.0 to 56.4)	56.4 (44.1 to 68.6)	62.5 (50.5 to 74.5)	58.5 (45.1 to 71.9)	5.7 (11.9 to 23.3)	0.53	61.7 (49.3 to 74.1)	64.6 (50.9 to 78.2)	-2.7 (-21.2 to 15.7)	0.77
**FECC-2**	In the last 6 months, did you know how to contact the person who helped you with managing your child's care when you needed help or had a question?	38.4 (28.2 to 55.7)	54.7 (42.4 to 67.0)	62.5 (50.5 to 74.5)	54.7 (41.2 to 68.3)	9.2 (-8.6 to 27.1)	0.31	60.0 (47.5 to 72.5)	60.4 (46.4 to 74.4)	-0.4 (-19.2 to 18.3)	0.97
**FECC-3**	Did the main provider/team help you to get the community services you or your child needed?	28.2 (18.7 to 51.8)	25.1 (14.4 to 35.7)	21.9 (11.6 to 32.1)	24.5 (12.1 to 36.2)	-3.5 (-17.9 to 10.9)	0.63	21.7 (11.2 to 32.2)	35.4 (21.8 to 49.1)	-13.9 (-30.7 to 2.8)	0.10
**FECC-4**	In the last 6 months did this person contact you without you getting in touch with them first?	22.8 (14.0 to 47.9)	28.2 (17.1 to 39.4)	45.3 (33.0 to 57.6)	34.0 (21.1 to 46.8)	11.4 (-6.3 to 29.1)	0.21	41.7 (29.1 to 54.2)	45.8 (31.6 to 60.1)	-4.1 (-22.9 to 0.7)	0.67
**FECC-5**	When the main provider/team contacted you, how often did they ask if you had any concerns about your child's health or treatment? When they contacted you, how often did they ask if your child's health had changed in any way?	17.3 (10.2 to 39.1)	20.2 (11.2 to 29.1)	35.1 (24.2 to 45.9)	25.1 (14.3 to 35.8)	10.0 (-5.2 to 25.1)	0.20	35.8 (24.5 to 47.0)	40.2 (27.2 to 53.3)	-4.5 (-21.4 to 12.5)	0.61
**FECC-6**	Has the main provider/team talked with you about the progress your child was making towards the goals written in their shared care plan?	3.2 (-0.4 to 19.6)	6.6 (0.4 to 12.8)	42.2 (30.0 to 54.4)	7.6 (0.4 to 14.7)	35.5 (20.9 to 50.0)	<0.001 *	36.7 (24.4 to 49.0)	43.8 (29.6 to 57.9)	-5.8 (-23.9 to 12.4)	0.54
**FECC-7**	Did the main provider/team contact you to make sure your child got an appointment to see a specialist?	23.4 (14.6 to 48.0)	38.1 (26.1 to 50.1)	26.6 (15.7 to 37.5)	30.2 (17.7 to 42.7)	0.1 (-15.5 to 15.7)	0.99	30.0 (18.3 to 41.7)	37.5 (23.7 to 51.3)	-8.6 (-26.6 to 9.4)	0.35

© 2023 Cohen E et al. *JAMA Pediatrics.*

		Baseline		Month 12				Month 24			
**Indicator ID [a]**	**Indicator Question**	**Intervention (N=77)**	**Waitlist (N=62)**	**Intervention (N=64)**	**Waitlist (N=53)**	**aMD (95% CI)**	**P Value**	**Intervention (N=60)**	**Waitlist (N=48)**	**aMD (95% CI)**	**P Value**
**FECC-8**	Did the main provider/ team know the important information about your child's health and care needs? Did they seem informed and up to date about the care your child got from other providers? Did they support your decisions about what is best for your child's health and treatment? Did they help you get appointments to visit other providers? Did they help you to get special medical equipment your child needed?	30.3 (22.4 to 43.5)	46.6 (36.0 to 57.3)	52.3 (41.9 to 62.8)	47.8 (36.3 to 59.3)	5.9 (-9.6 to 21.3)	0.46	52.9 (42.0 to 63.8)	55.5 (43.2 to 67.8)	-2.8 (-19.3 to 13.7)	0.74
**FECC-9**	How often did the written visit summaries you got from the main provider/team include a list of your child's health problems at the time of the visit? How often did they include an up-to-date list of all the prescription medicines your child is taking? Over the counter medicines? List of your child's allergies? Include all the names of all the specialist doctors who help care for your child? What to do if your child had a problem after the visit?	18.9 (12.2 to 36.7)	22.0 (13.5 to 30.6)	56.3 (46.2 to 66.3)	18.1 (9.2 to 27.0)	38.7 (25.4 to 52.1)	<0.001 *	54.0 (42.5 to 65.4)	49.2 (36.8 to 61.6)	5.0 (-11.6 to 21.7)	0.55
**FECC-10**	How often was the written visit summary you got from the main provider/team easy to understand? How often was it useful to you and your family?	23.3 (15.2 to 44.7)	26.2 (16.2 to 36.2)	62.9 (52.1 to 73.7)	23.6 (12.2 to 35.0)	39.5 (24.1 to 54.9)	<0.001 *	56.7 (44.9 to 68.5)	54.2 (40.8 to 67.6)	3.0 (-14.3 to 20.3)	0.73
**FECC-11**	The last time your child was in the hospital, did any of your child's doctors or nurses invite you to take part in hospital rounds?	62.7 (52.6 to 55.6)	63.3 (51.4 to 75.2)	73.4 (62.5 to 84.3)	77.4 (66.0 to 88.7)	2.7 (-18.3 to 12.8)	0.73	66.7 (54.7 to 78.7)	70.8 (57.8 to 83.8)	-1.2 (-17.7 to 15.2)	0.88

© 2023 Cohen E et al. *JAMA Pediatrics.*

		Baseline		Month 12				Month 24			
**Indicator ID [a]**	**Indicator Question**	**Intervention (N=77)**	**Waitlist (N=62)**	**Intervention (N=64)**	**Waitlist (N=53)**	**aMD (95% CI)**	**P Value**	**Intervention (N=60)**	**Waitlist (N=48)**	**aMD (95% CI)**	**P Value**
**FECC-12**	Did the written hospital stay summary include a list of all the health problems your child when leaving the hospital? Did it include a list of all the prescription medicines your child was taking? Over the counter medicines? List of your child's allergies? Names of all the specialist doctors who helped care for your child during the hospital stay? Did it include what the planned follow-up care was for your child after the hospital stay? Did it include who to call if your child had problems after the hospital stay?	45.6 (37.5 to 44.3)	46.4 (36.0 to 56.8)	33.9 (22.8 to 44.9)	47.8 (35.2 to 60.4)	-12.2 (-28.2 to 3.9)	0.14	29.7 (18.7 to 40.8)	34.2 (16.9 to 42.6)	-4.1 (-20.7 to 12.4)	0.62
**FECC-13**	Was the information in the written hospital stay summary you got easy to understand?	48.7 (39.8 to 48.6)	50.8 (39.0 to 62.6)	35.9 (24.3 to 47.6)	51.9 (38.4 to 65.4)	-13.8 (-30.8 to 3.2)	0.11	32.5 (20.7 to 44.3)	35.4 (21.8 to 49.1)	-2.0 (-19.5 to 15.5)	0.82
**FECC-14**	Did the main provider/team contact staff at your child's school to make sure they understood how your child's health conditions affected their ability to learn, understand or pay attention in class?	1.7 (-0.8 to 13.9)	4.9 (-0.4 to 10.3)	7.8 (1.2 to 14.4)	7.6 (0.4 to 14.7)	-0.9 (-10.5 to 8.8)	0.86	6.7 (0.3 to 13.0)	14.6 (4.5 to 24.7)	-7.2 (-18.7 to 4.3)	0.22
**FECC-16**	Has the main provider/team created a shared care plan for your child?	6.9 (1.9 to 27.4)	21.4 (11.2 to 31.6)	76.6 (66.1 to 87.0)	30.2 (17.7 to 42.7)	50.0 (34.8 to 65.1)	<0.001 *	78.3 (67.8 to 88.8)	75.0 (62.6 to 87.4)	0.9 (-15.3 to 17.2)	0.91
**FECC-17**	Has the main provider/team created an emergency care plan for your child?	13.3 (6.2 to 39.2)	11.7 (3.8 to 19.6)	37.5 (25.5 to 49.5)	11.3 (2.7 to 19.9)	21.5 (7.5 to 35.6)	0.003*	38.3 (25.9 to 50.7)	31.3 (18.0 to 44.5)	6.6 (-11.4 to 24.7)	0.47
**FECC-18**	Has the main provider/team created a written transition plan that summarized how your child's care will change and stay the same when they become an adult?	0.0 (0.0)	0.0 (0.0)	1.6 (-1.5 to 4.7)	0.0 (0.0)	1.6 (-1.8 to 4.9)	0.36	5.0 (-0.6 to 1-.6)	0.0 (0.0)	5.0 (-1.2 to 11.2)	0.11

All data are presented as mean (95% CI) unless otherwise indicated

© 2023 Cohen E et al. *JAMA Pediatrics.*

[a] 3 of the 20 FECC indicators were not collected (FECC-15, 19 and 20) as they had content related to: electronic health records (FECC-19 and 20), which were not readily available in the region of CCKO; and translation services (FECC-15), which was less relevant in a study population limited to English-speakers.
[b] P values were adjusted for multiple comparisons using the Holm-Bonferroni test where the overall target type 1 error rate was 0.05. Asterisk represents meeting significance threshold. Incorporating the Holm-Bonferroni correction, FECC-6, FECC-9, FECC-10, FECC-16 and FECC-17 were significantly different between the intervention and waitlist group at Year 1. No significant differences were observed at Year 2.

© 2023 Cohen E et al. *JAMA Pediatrics.*

eTable 6: Caregiver-reported secondary outcomes between the intervention and waitlist group at baseline, month 12 and month 24 follow-up after multiple imputation

	**Intervention**			**Waitlist**			**Month 12 [a]**		**Month 24 [b]**	
	**Baseline**	**Month 12**	**Month 24**	**Baseline**	**Month 12**	**Month 24**	**aMD (95% CI)**	**p-value [d]**	**aMD (95% CI)**	**p- value**
**Secondary Outcomes [c]**										
**Child measures**										
Quality of Life	47.8 (45.4 to 50.2)	55.1 (51.5 to 58.6)	53.4 (49.7 to 57.1)	50.1 (50.4 to 56.2)	51.6 (48.0 to 55.2)	52.8 (49.1 to 56.6)	4.9 (0.5 to 9.3)	0.03	2.1 (-2.7 to 7.0)	0.38
Emotional Health	41.7 (39.4 to 44.0)	47.4 (43.2 to 51.7)	45.8 (40.7 to 50.9)	39.1 (36.3 to 42.0)	44.6 40.4 to 48.9)	47.0 (41.5 to 52.6)	0.7 (-5.0 to 6.4)	0.80	-3.1 (-11.0 to 4.7)	0.42
Physical Pain	40.8 (35.4 to 46.2)	32.6 (25.1 to 40.1)	35.4 (26.7 to 44.1)	42.3 (49.4 to 35.2)	32.8 (25.5 to 40.1)	37.1 (28.2 to 46.0)	0.3 (-9.9 to 10.5)	0.95	-1.3 (-13.8 to 12.0)	0.83
**Parent measures**										
Physical Health	44.4 (42.7 to 46.1)	45.3 (43.0 to 47.7)	48.3 (45.6 to 51.0)	46.8 (44.6 to 49.0)	44.7 (42.2 to 47.2)	46.2 (43.5 to 48.9)	2.3 (-0.6 to 5.1)	0.12	3.5 (-0.4 to 7.4)	0.08
Mental Health	42.0 (39.9 to 44.1)	43.1 (40.5 to 45.6)	44.0 (40.9 to 47.0)	44.0 (41.2 to 46.8)	41.9 (39.3 to 44.6)	42.4 (39.5 to 45.4)	2.5 (-0.3 to 5.3)	0.07	2.6 (-1.7 to 6.9)	0.23
Satisfaction with Life	21.4 (19.9 to 22.9)	23.6 (21.7 to 25.5)	24.3 (22.2 to 26.5)	22.2 (20.2 to 24.2)	21.4 (19.3 to 23.6)	23.6 (20.8 to 26.5)	2.6 (0.2 to 5.0)	0.03	1.1 (-2.5 to 4.6)	0.55
Fatigue	57.1 (55.2 to 59.0)	55.2 (55.4 to 58.0)	54.4 (50.9 to 57.8)	56.4 (54.0 to 58.9)	58.1 (55.5 to 60.6)	57.1 (53.9 to 60.3)	-3.4 (-6.4 to -0.4)	0.03	-3.3 (-7.4 to 0.8)	0.12
Sleep Disturbance	57.1 (55.2 to 59.0)	55.4 (52.2 to 58.6)	54.4 (51.0 to 57.8)	57.5 (55.3 to 59.7)	58.8 (56.0 to 61.5)	59.2 (56.2 to 62.3)	-3.1 (-6.8 to 0.5)	0.09	-4.7 (-9.0 to -0.4)	0.03

Abbreviations: aMD, adjusted mean difference; CI, confidence interval
All data presented as mean (95% CI)
[a] aMD (95% CI), p value restricted to participants with completed baseline and month 12 follow up data
[b] aMD (95% CI), p value restricted to participants with completed baseline and month 24 follow up data
[c] aMD (95% CI), calculated using linear regression
[d] P values were adjusted for multiple comparisons using the Holm-Bonferroni test where the overall target type 1 error rate was 0.05. At 12 months the threshold for the secondary outcomes-child measures were 0.02 for quality of life, 0.03 for emotional health, and 0.05 for physical pain, for the parent measures were 0.05 for physical health, 0.01 for mental health, 0.02 for fatigue, 0.03 for sleep disturbance and 0.01 for satisfaction with life. At 24 months the threshold for the secondary outcomes-child measures were 0.03 for quality of life, 0.02 for emotional health, and 0.05 for physical pain, and for the parent measures were, 0.02 for physical health, 0.03 for mental health, 0.05 for satisfaction with life, 0.01 for fatigue, and 0.01 for sleep disturbance.

© 2023 Cohen E et al. *JAMA Pediatrics.*

eTable 7: Individual Family Experiences with Care Coordination (FECC) outcomes baseline, month 12 and month 24 follow-up after multiple imputation

		**Baseline**		**Month 12**				**Month 24**			
**Indicator ID [a]**	**Indicator Question**	**Intervention (N=77)**	**Waitlist (N=62)**	**Intervention (N=64)**	**Waitlist (N=53)**	**aMD (95% CI)**	**P Value [b]**	**Intervention (N=60)**	**Waitlist (N=48)**	**aMD (95% CI)**	**P Value**
**FECC-1**	Did the main provider/team help you to manage your child's care or treatment from different doctors and care providers?	41.3 (31.0 to 56.4)	56.4 (44.1 to 68.6)	57.4 (42.4 to 72.4)	59.3 (45.7 to 72.9)	0.2 (-19.9 to 20.2)	0.99	56.8 (39.9 to 73.7)	64.1 (47.8 to 80.4)	-7.2 (-31.8 to 17.4)	0.57
**FECC-2**	In the last 6 months, did you know how to contact the person who helped you with managing your child's care when you needed help or had a question?	38.4 (28.2 to 55.7)	54.7 (42.4 to 67.0)	56.8 (41.8 to 71.7)	55.1 (41.4 to 68.8)	3.0 (-17.4 to 23.3)	0.77	54.8 (36.6 to 72.9)	58.9 (41.7 to 76.0)	-3.6 (-29.4 to 22.2)	0.78
**FECC-3**	Did the main provider/team help you to get the community services you or your child needed?	28.2 (18.7 to 51.8)	25.1 (14.4 to 35.7)	19.8 (7.3 to 32.2)	23.1 (11.7 to 34.6)	-4.4 (20.0 to 11.2)	0.58	21.4 (5.9 to 36.8)	34.0 (19.3 to 48.6)	-12.6 (34.4 to 9.1)	0.25
**FECC-4**	In the last 6 months did this person contact you without you getting in touch with them first?	22.8 (14.0 to 47.9)	28.2 (17.1 to 39.4)	42.3 (27.0 to 57.5)	33.2 (19.8 to 46.6)	9.4 (-10.5 to 29.3)	0.35	39.0 (22.4 to 55.6)	45.9 (29.2 to 62.6)	6.8 (-31.0 to 17.5)	0.58
**FECC-5**	When the main provider/team contacted you, how often did they ask if you had any concerns about your child's health or treatment? When they contacted you, how often did they ask if your child's health had changed in any way?	17.3 (10.2 to 39.1)	20.2 (11.2 to 29.1)	33.3 (20.1 to 46.5)	23.0 (11.1 to 34.8)	9.8 (-7.5 to 27.1)	0.27	33.4 (18.1 to 48.6)	38.9 (23.9 to 54.0)	-5.8 (-27.9 to 16.3)	0.60
**FECC-6**	Has the main provider/team talked with you about the progress your child was making towards the goals written in their shared care plan?	3.2 (-0.4 to 19.6)	6.6 (0.4 to 12.8)	40.4 (26.6 to 54.2)	8.4 (-1.2 to 17.9)	33.6 (17.2 to 50.0)	<0.001*	36.0 (18.4 to 53.6)	43.8 (25.9 to 61.7)	-5.8 (-31.6 to 20.1)	0.66
**FECC-7**	Did the main provider/team contact you to make sure your child got an appointment to see a specialist?	23.4 (14.6 to 48.0)	38.1 (26.1 to 50.1)	24.7 (11.2 to 38.1)	31.2 (18.5 to 43.8)	-2.0 (-19.8 to 15.8)	0.83	28.0 (12.9 to 43.2)	37.2 (21.4 to 53.1)	-9.2 (-31.6 to 13.1)	0.42

© 2023 Cohen E et al. *JAMA Pediatrics.*

		Baseline		Month 12				Month 24			
**Indicator ID [a]**	**Indicator Question**	**Intervention (N=77)**	**Waitlist (N=62)**	**Intervention (N=64)**	**Waitlist (N=53)**	**aMD (95% CI)**	**P Value**	**Intervention (N=60)**	**Waitlist (N=48)**	**aMD (95% CI)**	**P Value**
**FECC-8**	Did the main provider/ team know the important information about your child's health and care needs? Did they seem informed and up to date about the care your child got from other providers? Did they support your decisions about what is best for your child's health and treatment? Did they help you get appointments to visit other providers? Did they help you to get special medical equipment your child needed?	30.3 (22.4 to 43.5)	46.6 (36.0 to 57.3)	48.5 (35.4 to 61.5)	48.0 (36.2 to 59.7)	2.0 (-15.7 to 19.6)	0.83	49.4 (34.5 to 64.3)	54.9 (41.2 to 68.7)	-6.3 (-27.7 to 15.2)	0.56
**FECC-9**	How often did the written visit summaries you got from the main provider/team include a list of your child's health problems at the time of the visit? How often did they include an up-to-date list of all the prescription medicines your child is taking? Over the counter medicines? List of your child's allergies? Include all the names of all the specialist doctors who help care for your child? What to do if your child had a problem after the visit?	18.9 (12.2 to 36.7)	22.0 (13.5 to 30.6)	53.0 (40.9 to 65.2)	20.4 (10.7 to 30.1)	33.2 (17.9 to 48.5)	<0.001*	55.2 (38.1 to 72.4)	50.1 (35.3 to 65.0)	5.6 (-17.4 to 28.6)	0.63
**FECC-10**	How often was the written visit summary you got from the main provider/team easy to understand? How often was it useful to you and your family?	23.3 (15.2 to 44.7)	26.2 (16.2 to 36.2)	58.9 (45.8 to 72.0)	26.0 (14.1 to 37.9)	33.3 (16.1 to 50.4)	<0.001*	57.2 (39.3 to 75.0)	55.3 (39.8 to 70.9)	2.3 (-21.7 to 26.2)	0.85
**FECC-11**	The last time your child was in the hospital, did any of your child's doctors or nurses invite you to take part in hospital rounds?	62.7 (52.6 to 55.6)	63.3 (51.4 to 75.2)	71.8 (58.4 to 85.2)	78.7 (66.9 to 90.4)	-6.8 (-24.3 to 10.7)	0.45	65.3 (48.1 to 82.4)	72.5 (58.0 to 87.1)	-7.0 (-29.5 to 15.4)	0.53

© 2023 Cohen E et al. *JAMA Pediatrics.*

		Baseline		Month 12				Month 24			
**Indicator ID [a]**	**Indicator Question**	**Intervention (N=77)**	**Waitlist (N=62)**	**Intervention (N=64)**	**Waitlist (N=53)**	**aMD (95% CI)**	**P Value**	**Intervention (N=60)**	**Waitlist (N=48)**	**aMD (95% CI)**	**P Value**
**FECC-12**	Did the written hospital stay summary include a list of all the health problems your child when leaving the hospital? Did it include a list of all the prescription medicines your child was taking? Over the counter medicines? List of your child's allergies? Names of all the specialist doctors who helped care for your child during the hospital stay? Did it include what the planned follow-up care was for your child after the hospital stay? Did it include who to call if your child had problems after the hospital stay?	45.6 (37.5 to 44.3)	46.4 (36.0 to 56.8)	35.1 (22.4 to 47.7)	50.9 (37.9 to 63.9)	-15.6 (-32.8 to 1.6)	0.08	30.4 (14.8 to 46.0)	31.0 (15.3 to 46.7)	-0.4 (-22.9 to 22.1)	0.97
**FECC-13**	Was the information in the written hospital stay summary you got easy to understand?	48.7 (39.8 to 48.6)	50.8 (39.0 to 62.6)	37.3 (23.9 to 50.7)	54.0 (40.3 to 67.7)	-16.1 (-34.3 to 2.1)	0.08	34.6 (14.3 to 55.0)	32.1 (15.7 to 48.5)	3.1 (-20.1 to 26.3)	0.79
**FECC-14**	Did the main provider/team contact staff at your child's school to make sure they understood how your child's health conditions affected their ability to learn, understand or pay attention in class?	1.7 (-0.8 to 13.9)	4.9 (-0.4 to 10.3)	4.3 (-3.7 to 12.3)	7.7 (0.5 to 14.9)	-4.4 (-14.7 to 6.0)	0.41	12.2 (1.5 to 23.0)	17.9 (6.9 to 29.0)	-5.7 (-20.3 to 8.8)	0.44
**FECC-16**	Has the main provider/team created a shared care plan for your child?	6.9 (1.9 to 27.4)	21.4 (11.2 to 31.6)	71.0 (58.2 to 83.8)	33.8 (21.0 to 46.6)	42.2 (25.0 to 59.3)	<0.001*	69.3 (53.2 to 85.4)	78.3 (64.4 to 92.2)	-10.9 (-33.1 to 11.2)	0.33
**FECC-17**	Has the main provider/team created an emergency care plan for your child?	13.3 (6.2 to 39.2)	11.7 (3.8 to 19.6)	33.6 (19.6 to 47.6)	13.5 (3.8 to 23.2)	16.6 (0.2 to 33.1)	0.05	36.2 (14.3 to 58.0)	31.9 (16.3 to 47.4)	4.0 (-23.2 to 31.2)	0.8
**FECC-18**	Has the main provider/team created a written transition plan that summarized how your child's care will change and stay the same when they become an adult?	0 (0)	0 (0)	1.4 (-1.7 to 4.5)	0.2 (-1.5 to 2.0)	1.1 (-2.7 to 4.8)	0.57	4.2 (-1.4 to 9.8)	1.6 (-3.4 to 6.7)	2.5 (-4.7 to 9.7)	0.49

© 2023 Cohen E et al. *JAMA Pediatrics.*

All data are presented as mean (95% CI) unless otherwise indicated

[a] 3 of the 20 FECC indicators were not collected (FECC-15, 19 and 20) as they had content related to: electronic health records (FECC-19 and 20), which were not readily available in the region of CCKO; and translation services (FECC-15), which was less relevant in a study population limited to English-speakers.

[b] Asterisk represents meeting significance threshold based on Holm-Bonferroni correction.

© 2023 Cohen E et al. *JAMA Pediatrics.*

eTable 8: Health service cost and utilization among study participants using the initial complex care clinic visit date as the start of the intervention.

	Year 1				Year 2			
**Variable**	**Intervention** (N=79)	**Waitlist** (N=62) [a]	**MD**	**P Value**	**Intervention** (N=75)	**Waitlist** (N=59)	**MD** [c]	**P Value**
**Health Service Cost ($)**								
	46,396 (17,374-95,165)	50,176 (24,188, 120,122)	-3780 (-31653.6 to 26742)	0.31	12,226 (3,499-53,467)	37,524 (9,338, 119,547)	-25298 (-52182.6 to 1586.6)	<0.001
**Health Service Utilization**								
**Admissions**	1 (0-3)	2 (0-4)	-1 (-1.9 to 0.1)	0.68	1 (0-3)	1 (0-3)	0 (-0.9 to 0.9)	0.67
**Inpatient LOS**	3 (0-14)	3 (0-16)	0 (-4.5 to 4.5)	0.38	0 (0-8)	2 (0-9)	-2 (-4.2 to 0.2)	0.22
**ICU LOS** [b]	2 (1-4)	3 (1-7)	-1 (-7.2 to 5.2)	0.77	3 (1-5)	9 (3-10)	-6 (-12.4 to 0.4)	0.12
**ED visits**	2 (1-4)	2 (1-4)	0 (-0.9 to 0.9)	0.61	1 (0-2)	1 (0-4)	0 (-0.9 to 0.9)	0.11
**Primary care visits**	2 (1-6)	3 (1-7)	-1 (-2.8 to 0.8)	0.33	1 (0-3)	1 (0-3)	0 (-0.9 to 0.9)	0.39
**Specialist visits**	13 (10-17)	13 (8-20)	0 (-3.4 to 3.4)	0.92	11 (6-17)	12 (8-17)	-1 (-4.8 to 2.8)	0.37
**Prescriptions**	25 (14-52)	30 (15-67)	-5 (-22.2 to 12.2)	0.20	25 (7-45)	28 (11-69)	-3 (-20.0 to 14.0)	0.20
**Home Care, No. (%)**	75 (95)	52 (84)	11.1% (1.2% to 20.9%)	0.03	64 (85)	53 (90)	-4.5% (-15.9% to 6.9%)	0.44

Abbreviations: MD, median difference; CI, confidence interval; LOS, length of stay; ICU, intensive care unit; ED, emergency department
All data are presented as median (IQR); unless otherwise indicated.
[a] 1 participant died before their first complex care clinic visit
[b] ICU LOS calculated amongst those with an ICU stay. In Year 1, 16 participants in the intervention group and 13 in the waitlist group had an ICU stay. In Year 2, 12 participants in the intervention group and 9 in the waitlist group had an ICU stay.
[c] MD calculated as the difference between intervention and waitlist groups. P-values were calculated using Kruskal-Wallis tests for medians and $\chi 2$ for proportions.

© 2023 Cohen E et al. *JAMA Pediatrics.*

eTable 9: Health service cost and utilization among study participants who had their entire Year 1 and Year 2 follow-up before estimated onset date of the pandemic (March 13, 2020).

	Year 1				Year 2			
**Variable**	**Intervention** (N=68)	**Waitlist** (N=56)	**MD**	**P Value**	**Intervention** (N=65)	**Waitlist** (N=53)	**MD** [b]	**P Value**
**Health Service Cost ($)**								
	45,927 (20,013-110,926)	52,843 (27,098-127,440)	-6916 (-39883.4 to 28437.4)	0.62	21,318 (8,707-71,177)	40,661 (20,896-119,547)	-19343 (-51692.8 to 13006.8)	0.03
**Health Service Utilization**								
**Admission**	2 (1-4)	2 (1-4)	0 (-10.9 to 10.9)	0.80	1 (0-3)	1 (0-3)	0 (-1.2 to 1.2)	0.63
**Inpatient LOS**	4 (0-18)	6 (1-17)	-2 (-8.4 to4.4)	0.57	1 (0-9)	2 (0-9)	-1 (-3.8 to 1.8)	0.28
**ICU LOS** [a]	2 (1-14)	3 (1-7)	1 (-7.3 to 7.3)	0.83	3 (1-5)	9 (3-10)	-5 (-12.5 to 2.5)	0.14
**ED visits**	2 (1-4)	2 (1-5)	0 (-1.4 to 1.4)	0.99	1 (0-2)	2 (0-4)	-1 (-2.5 to 0.5)	0.24
**Primary care visits**	3 (1-6)	3 (1-7)	0 (-1.6 to 1.6)	0.77	2 (1-4)	1 (1-3)	1 (0.1 to 1.9)	0.24
**Specialist visits**	16 (11-20)	13 (8-20)	3 (-0.4 to 6.4)	0.20	11 (7-17)	12 (9-17)	-1 (-4.7 to 2.7)	0.30
**Prescriptions**	27 (16-45)	29 (11-67)	-2 (-19.8 to 15.8)	0.58	24 (5-41)	27 (9-68)	-3 (-22.4 to 16.4)	0.30
**Home care, No. (%)**	64 (94)	47 (84)	10.2% (-0.6% to 21.0%)	0.07	58 (89)	47 (89)	0.6% (-10.9% to 12.0%)	0.92

Abbreviations: MD, median difference; CI, confidence interval; LOS, length of stay; ICU, intensive care unit; ED, emergency department
All data are presented as median (IQR) unless otherwise indicated.
[a] ICU LOS calculated amongst those with an ICU stay. In year 1, 18 participants in the intervention group and 14 in the waitlist group had an ICU stay. In year 2, 10 participants in the intervention group and 12 in the waitlist group had an ICU stay.
[b] MD calculated as the difference between intervention and waitlist groups. P-values were calculated using Kruskal-Wallis tests for medians and $\chi2$ for proportions.

© 2023 Cohen E et al. *JAMA Pediatrics.*

eTable 10: Health service cost and utilization among study participants, excluding deaths in Year 1 and Year 2.

	Year 1 [a]				Year 2 [b]			
**Variable**	**Intervention** (N=76)	**Waitlist** (N=59)	**MD**	**P Value**	**Intervention** (N=75)	**Waitlist** (N=58)	**MD** [c]	**P Value**
**Health Service Cost ($)**								
	48,509 (20,268-101,238)	48,852 (24,188-134,757)	-343 (-28963.0 to 28357.0)	0.68	17,331 (5,797-61,429)	37,382 (9,338-119,547)	-20,051 (-48242.8 to 8424.8)	0.02
**Health Service Utilization**								
**Admissions**	2 (1-4)	2 (0-4)	0 (-0.9 to 0.9)	0.98	1 (0-2)	1 (0-3)	0 (-0.9 to 0.9)	0.66
**Inpatient LOS**	4 (0-12)	3 (0-16)	1 (-3.6 to 5.6)	0.76	1 (0-5)	2 (0-8)	0 (-2.2 to 2.2)	0.24
**ICU LOS** [c]	2 (1-7)	3 (1-7)	-1 (-8.3 to 6.3)	0.85	3 (1-5)	9 (3-13)	-6 (-11.4 to 0.4)	0.13
**ED visits**	2 (1-4)	2 (1-4)	0 (-0.9 to 0.9)	0.81	1 (0-2)	1 (0-4)	0 (-1.2 to 1.2)	0.26
**Primary care visits**	3 (1-6)	3 (1-7)	0 (-1.6 to 1.6)	0.87	2 (0-4)	1 (0-3)	1 (0.1 to 1.9)	0.67
**Specialist visits**	15 (11-20)	13 (8-20)	2 (-1.2 to 5.2)	0.30	11 (7-17)	12 (9-17)	-1 (-4.4 to 2.4)	0.37
**Prescriptions**	27 (15-45)	35 (11-67)	-8 (-24.6 to 8.6)	0.50	24 (5-49)	28 (12-69)	-4 (-22.2 to 14.2)	0.19
**Home care, No. (%)**	72 (95)	49 (83)	11.7% (1.4% to 21.9%)	0.03	67 (89)	52 (90)	-0.3% (-10.9% to 10.3%)	0.95

Abbreviations: MD, median difference; CI, confidence interval; LOS, length of stay; ICU, intensive care unit; ED, emergency department
All data are presented as median (IQR) unless otherwise indicated.
[a] total N=135 for Year 1 analysis as 6 participants (3 in intervention group and 3 in waitlist group) died during Year 1.
[b] total N=133 for Year 2 analysis as 2 participants (1 in intervention and 1 in waitlist group) died during Year 2.
[c] ICU LOS calculated amongst those with an ICU stay. In Year 1, 18 participants in the intervention group and 14 in the waitlist group had an ICU stay. In Year 2, 11 participants in the intervention group and 11 in the waitlist group had an ICU stay.
[c] MD calculated as the difference between intervention and waitlist groups. P-values were calculated using Kruskal-Wallis tests for medians and $\chi 2$ for proportions.

© 2023 Cohen E et al. *JAMA Pediatrics.*

eTable 11: Health service cost and utilization among study participants who had their entire 1st and 2nd year follow-up before estimated onset date of the pandemic (March 13, 2020), excluding deaths in Year 1 and Year 2.

	Year 1				Year 2			
**Variable**	**Intervention** (N=65)	**Waitlist** (N=53)	**MD**	**P Value**	**Intervention** (N=64)	**Waitlist** (N=52)	**MD [b]**	**P Value**
**Health Service Cost ($)**								
	45,609 (20,009-107,259)	53,760 (27,116-134,757)	-8151 (-43652 to 27350)	.41	22,117 (8,026-77,720)	39,517 (19,537-127,871)	-17,400 (-47920, 17006)	.04
**Health Service Utilization**								
Admissions	1 (1-4)	2 (1-4)	-1 (-1.9 to -0.1)	.62	1 (0-2)	1 (0-3)	0 (-1.2 to 1.2)	.57
Inpatient LOS	4 (0-18)	6 (1-17)	-2 (-7.4 to 3.4)	.44	1 (0-7)	2 (0-8)	-1 (-3.6 to 1.6)	.27
ICU LOS [a]	3 (1-7)	3 (1-7)	0 (-7.7 to 7.7)	.97	3 (1-5)	9 (3-13)	-5 (-12.6 to 2.6)	.18
ED visits	2 (1-5)	2 (1-5)	0 (-1.4 to 1.4)	.83	1 (0-2)	2 (0-4)	-1 (-2.5 to 0.5)	.23
Primary care visits	3 (1-7)	3 (1-7)	0 (-2.1 to 2.1)	.83	2 (1-4)	1 (1-3)	1 (0.1 to 1.9)	.27
Specialist visits	16 (11-20)	13 (8-20)	3 (-0.6 to 6.6)	.25	11 (7-17)	12 (9-17)	-1 (-4.7 to 2.7)	.26
Prescriptions	25 (16-45)	29 (11-67)	-4 (-22.4 to 14.4)	.56	24 (5-41)	27 (9-68)	-2 (-20.7 to 16.7)	.29
Home Care, No. (%)	61 (94)	44 (83)	10.8% (-0.5% to 22.1%)	.06	57 (89)	46 (89)	0.6% (-11.0% to-2.2%)	.92

Abbreviations: MD, median difference; CI, confidence interval; LOS, length of stay; ICU, intensive care unit; ED, emergency department
All data are presented as median (IQR) unless otherwise indicated.
[a] ICU LOS calculated amongst those with an ICU stay. In Year 1, 16 participants in the intervention group and 14 in the waitlist group had an ICU stay. In Year 2, 10 participants in the intervention group and 11 in the waitlist group had an ICU stay.
[b] MD calculated as the difference between intervention and waitlist groups. P-values were calculated using Kruskal-Wallis tests for medians and $\chi2$ for proportions.

© 2023 Cohen E et al. *JAMA Pediatrics.*
