# Supplementary material for: Effectiveness of Structured Care Coordination for Children With Medical Complexity: The Complex Care for Kids Ontario (CCKO) Randomized Clinical Trial
Source: JAMA Pediatr. 2023 Mar 20;177(5):461–71. doi: 10.1001/jamapediatrics.2023.0115 (PMC10028546; doi:10.1001/jamapediatrics.2023.0115)
Supplement: Supplement 3. — Data sharing statement [file jamapediatr-e230115-s003.pdf]

Data Sharing Statement

Population-level Implementation of Care Coordination for Children with Medical Complexity: The Complex Care for Kids Ontario (CCKO) Randomized Clinical Trial

Published: March 20, 2023
doi:10.1001/jamapediatrics.2023.0115

Data

Data available: Upon request
Data types: Deidentified participant data
How to access data: The data sets from this study are held securely in coded form at ICES. Data-sharing agreements prohibit ICES from making the data sets publicly available, but access may be granted to those who meet pre-specified criteria for confidential access, available at www.ices.on.ca/DAS
When available:

Supporting Documents

Document types: None

Additional Information

Who can access the data: Researchers who have signed a data access agreement and had their proposed use of the data approved.
Types of analyses: For a specified purpose approved by study authors.
Mechanisms of data availability: Upon proposal acceptance and a signed data access agreement.
