# Supplementary material for: Effectiveness of Structured Care Coordination for Children With Medical Complexity: The Complex Care for Kids Ontario (CCKO) Randomized Clinical Trial
Source: JAMA Pediatr. 2023 Mar 20;177(5):461–71. doi: 10.1001/jamapediatrics.2023.0115 (PMC10028546; doi:10.1001/jamapediatrics.2023.0115)
Supplement: Supplement 4. — Protocol changes [file jamapediatr-e230115-s004.pdf]

Protocol: Complex Care for Kids Ontario (CCKO): A patient- and family-centred implementation and evaluation of care coordination for children with medical complexity

An amendment has been drafted to the CCKO study protocol. The following is a summary of key changes to the previous version of the protocol (version date: February 14, 2022) including the rationale for each change.

Original Text	Modified Text	Rationale
Andy Willan listed as study statistician	Anna Health added as second study statistician	Retirement of Dr. Willan
"The distributions for each of the outcomes will be examined, and if approximately normally distributed, each outcome variable at Month 12 will be compared between groups using an analysis of covariance with the corresponding baseline score as the covariate."	Each outcome variable at Month 12 will be compared between groups using an ordinal regression incorporating center as a random intercept and adjusting for the baseline score.	The outcome for the FECC are ordinal by nature. An ordinal regression is more appropriate. Center effects need to be accommodated in the analysis.
No analytic plan for missing data	Additional sensitivity analysis: "In addition to a complete case analysis, since it was noted on original analysis that there was substantial missing data, the data were analyzed imputing missing values. Missing primary and secondary outcome data were imputed using multiple imputation by chained equations incorporating baseline variables, group assignment and the corresponding outcomes at baseline and 6 months."	Missing data not accounted for in any of the original analyses.
